# Antibiotics induce polarization of pleural macrophages to M2-like phenotype in patients with tuberculous pleuritis

**DOI:** 10.1038/s41598-017-14808-9

**Published:** 2017-11-03

**Authors:** Sisi Wang, Jian Zhang, Liyan Sui, Hao Xu, Qianling Piao, Ying Liu, Xinglong Qu, Ying Sun, Lei Song, Dan Li, Liping Peng, Shucheng Hua, Guangan Hu, Jianzhu Chen

**Affiliations:** 1 Department of Translational Medicine, The First Hospital, Jilin University, Changchun, 130061 China; 2 The Infectious Disease Hospital of Changchun, Changchun, Jilin, Changchun, 130123 China; 3 Department of Respiratory Medicine, The First Hospital, Jilin University, Changchun, 130021 China; 4 Koch Institute for Integrative Cancer Research and Department of Biology, Massachusetts Institute of Technology, 77 Massachusetts Avenue, Cambridge, Massachusetts 02139 USA

## Abstract

Pleural macrophages play critical roles in pathogenesis of tuberculous pleuritis, but very little is known about their response to anti-tuberculosis antibiotics treatment. Here, we examined whether and how pleural macrophages change in phenotype, transcription and function following antibiotics treatment in patients with tuberculous pleuritis. Results show pro-inflammatory cytokines were down-regulated significantly post antibiotic treatment in the pleural effusions and pleural macrophages up-regulated markers characteristic of M2 macrophages such as CD163 and CD206. Differential expression analysis of transcriptomes from four paired samples before and after treatment identified 230 treatment-specific responsive genes in pleural macrophages. Functional analysis identified interferon-related pathway to be the most responsive genes and further confirmed macrophage polarization to M2-like phenotype. We further demonstrate that expression of a significant fraction of responsive genes was modulated directly by antibiotics in pleural macrophages *in vitro*. Our results conclude that pleural macrophages polarize from M1-like to M2-like phenotype within a mean of 3.5 days post antibiotics treatment, which is dependent on both pleural cytokine environment and direct modulatory effects of antibiotics. The treatment-specific genes could be used to study the roles of pleural macrophages in the pathogenesis of tuberculous pleuritis and to monitor the response to antibiotics treatment.

## Introduction

Tuberculosis (TB) is caused by infection of *Mycobacterium tuberculosis* in the lung and is a major public health problem in the world because of its prevalence and high mortality^1^. Around 20% of TB patients develop tuberculous pleural effusion (TPE), an accumulation of excess fluid in the pleural cavity, due to tuberculous pleuritis, i.e., inflammation of the pleura^2–4^. Tuberculous pleuritis usually presents as an acute illness with fever, cough and pleuritic chest pain, and is diagnosed by elevated levels of adenosine deaminase (ADA) and interferon-gamma (IFNγ) in the pleural fluid^5,6^. Tuberculous pleuritis is caused by delayed type hypersensitivity responses to mycobacterial antigens in the pleural space with low bacterial burden^7^. Although ~70% pleural fluid cells are T cells, pleural macrophages are also known to contribute to the pathogenesis of tuberculous pleuritis and the outcome of different clinic forms of TB^8,9^.

Macrophages are the first line of defense against invading microbes and mediate innate immune responses through pathogen recognition and activation of inflammatory reactions. Depending on the stimulations and microenvironment, macrophages polarize to different functional states, including the classically activated M1 and the alternatively activated M2^10^. The M1 phenotype is associated with secretion of large quantities of pro-inflammatory cytokines and killing of microbes. The M2 type of macrophages exhibits immunoregulatory, phagocytosis, and tissue remodeling and repair functions. In TB patients, alveolar macrophages in the lungs are directly infected by *M*. *tuberculosis*. Depending on the stage of infection and disease development, macrophages can produce either pro-inflammatory and microbicidal responses or immunosuppressive and tissue repairing responses^11^. For example, in newly onset TB patients and during the early phase of *M*. *tuberculosis* infection, macrophages exhibit an M1-like phenotype producing pro-inflammatory cytokines and contributing to the development of TPE^8^.

The frontline treatment of *M*. *tuberculosis* infection is a combination of four antibiotics: isoniazid, rifampicin, pyrazinamide and ethambutol (HRZE). Antibiotics treatment significantly eliminates the bacterial load and inhibits the formation of TPE. Although antibiotics are most widely known for their direct microbicidal activity^12,13^, increasing evidence suggests that many antibiotics also modulate activities of immune cells^14^. For example, rifampicin is known to induce the expression of CD1 in human T cells and monocytes and therefore immune responses to various infections^15,16^. Isoniazid eliminates antigen-specific T cells by inducing apoptosis in TB animal models^17^, and also regulates both adaptive and innate immune responses during its induction of liver injury^18^. Pyrazinamide modulates the host immune response to infection by reducing pro-inflammatory cytokines IL6 and TNFα production^19^, and activates the mouse macrophage by inducing expression of CD80, CD86 and MHCII^20^. Isoniazid and pyrazinamide together robustly activate autophagy through reactive oxygen species in mycobacterial-infected murine macrophages^21^. A pertinent question is whether and how antibiotics treatment in TB patients modulates macrophage polarization and function.

Recently, O’Garra and colleagues have started to address this question by transcriptional profiling of peripheral blood mononuclear cells (PBMCs) from TB patients before and after antibiotics treatment. They report the identification of a whole-blood host-response-specific 393-transcript signature, which reflects mycobacterial infection, and a whole-blood treatment-specific 320-transcript signature for monitoring the efficiency of antibiotics treatment^22,23^. Because total PBMCs were used, their studies did not define the contribution of specific immune cell types. Furthermore, compared to TPE, although peripheral blood is more accessible but it is farther removed from the primary site of mycobacterial infection. Whether and how antibiotics treatment in TB patients affects specific immune cell types, such as macrophages, in the proximal site of infection has not been investigated.

In this study we have examined changes in phenotype and function of pleural macrophages in TB patients before and after antibiotics treatment by assaying their expression of surface markers, transcription profiles and phagocytosis activities. Our results show that pleural macrophages are polarized from M1-like to M2-like phenotype as early as 2 days post antibiotics treatment. A signature of 230 genes was identified to reflect the treatment-specific response in pleural macrophages. Among these genes, some were modulated by direct antibiotics treatment of purified pleural macrophages *in vitro*. Our study defines responses of pleural macrophages to antibiotics treatment in TB patients and raises the possibility of therapeutic efficacy of antibiotics in treating tuberculous pleuritis by modulating activities of pleural macrophages.

## Results

### Pleural macrophages are polarized in response to antibiotics treatment

**Figure 1:**
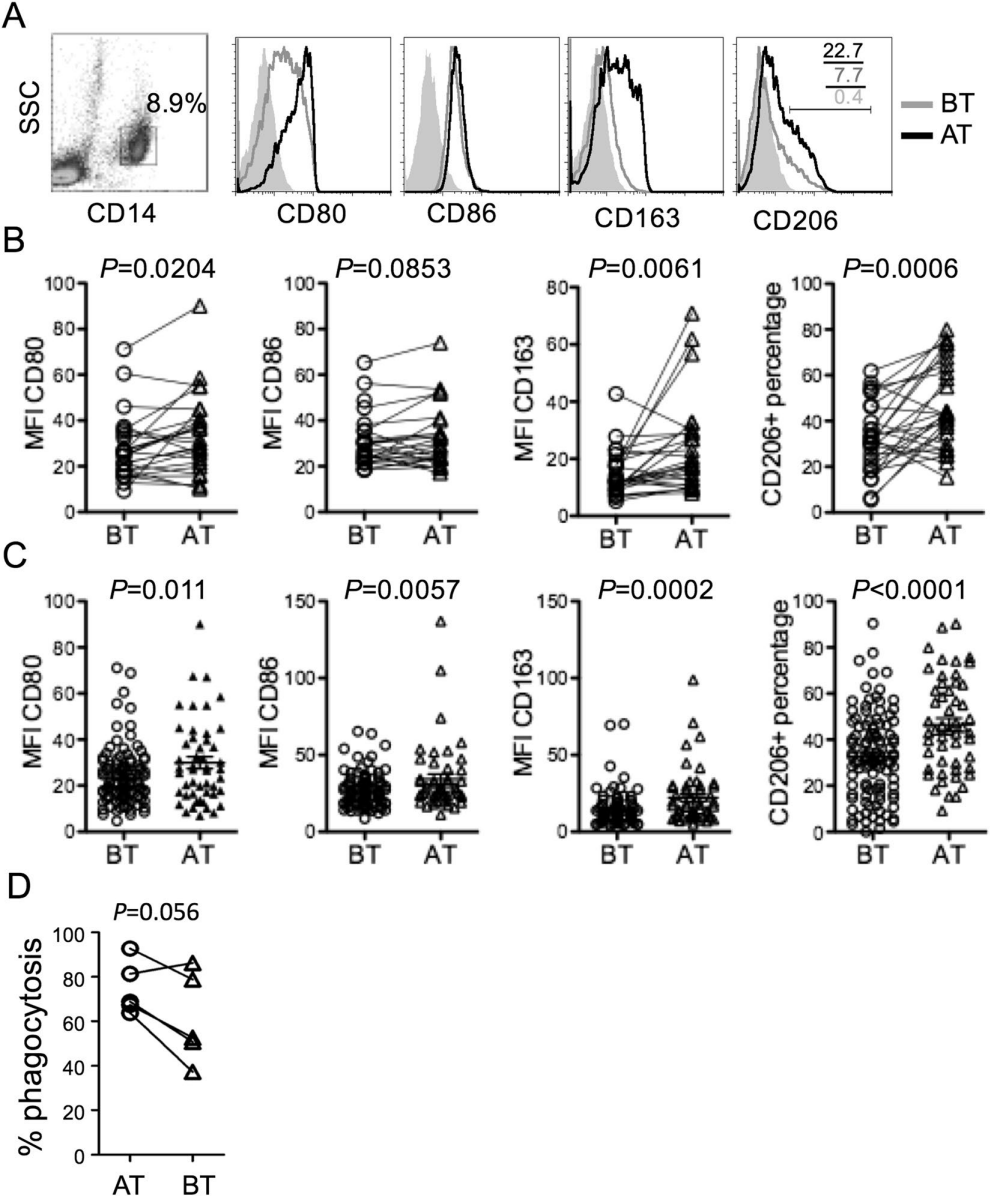
Comparison of surface marker expression by pleural macrophages before and after antibiotics treatment. (**A**) Flow cytometry analysis of CD80, CD86, CD163 and CD206 expression by CD14^+^ pleural macrophages before (BT) and after (AT) antibiotics treatment from a representative patient. Shaded histograms show cells stained with isotype control antibody. The numbers indicate percentages of cells within the gated regions. (**B**–**C**) Aggregated data from paired samples (B, n = 29) and unpaired samples (C, n = 128) showing mean fluorescence intensity (MFI) of CD80, CD86 and CD163 expression and percentages of CD206^+^ cells. P values are shown between BT and AT. (**D**) Comparison of phagocytosis of five paired pleural macrophage samples before and after antibiotics treatment.

We enrolled 171 patients with tuberculous pleuritis for studying the effect of antibiotics on macrophage polarization. After excluding patients with other lung diseases, HIV co-infection, or contraindications to pleural effusion puncture, 135 patients remained (Table 1). Pleural fluids were collected from 36 patients before and 2 to 10 days after HRZE treatment (paired samples). In the remaining 99 patients, pleural fluids were collected either before or after treatment only (unpaired samples). The frequency and phenotype of various immune cells in the pleural fluids were determined by flow cytometry using commonly used lymphocyte and macrophage markers (Figs S1 and 1A). Overall, CD3^+^ T cells were most abundant in the pleural fluids with a CD4^+^ to CD8^+^ ratio of ~3 to 1, similar to a previous report^24^. The proportion of macrophages in the pleural fluids varied from patients to patients with an average of 8.5% (Table 1). The frequencies of CD4^+^ or CD8^+^ T cells, B cells, nature killer cells (NK) and macrophages did not change significantly before and after antibiotics treatment in the paired and unpaired analysis (Figure S2). However, expression of M2 markers, including CD163 and CD206, was significantly elevated in pleural macrophages in both paired (Fig. 1B) and unpaired (Fig. 1C) samples following antibiotics treatment, although expression of M1 markers CD80 and CD86 were also increased slightly. The phagocytic activities of pleural macrophages to engulf fluorescent beads were decreased following antibiotics treatment with a *P*-value of < 0.056 (Fig. 1D). These data suggest that pleural macrophages are polarized following antibiotics treatment in patients with tuberculous pleuritis.

**Table 1:** Clinical characteristics of patients.

Characteristics	Statistics
Male/Female	89/46
Ages (years)	35.6 (17~83)
Pleural fluid	
WBC (6 × 10^9^/L)	4.5 (0.6~23)
Mononecluear cells (%)	72.3 (39~94)
CD4+	58.6 (29.8~82.3)
CD8+	19.5 (6.9~34.6)
B	5.7 (0.3~26.9)
NK	6.3 (0.4~21.4)
Macrophages	8.5 (0.2~53.6)
Multinuclear cells (%)	27.7 (6~61)
ADA (U/L)	84.9 (37~145)
LDH (U/L)	782.2 (199~1462)
Protein (g/L)	52.2 (26~88)
Rivalta test	+
Serum	
ESR (mm/h)	48.7 (8~76)
CRP (mg/L)	65.7 (19~121.8)
CDU	66.6 (27~123)

### Antibiotics inhibit pro-inflammatory cytokine production

**Figure 2:**
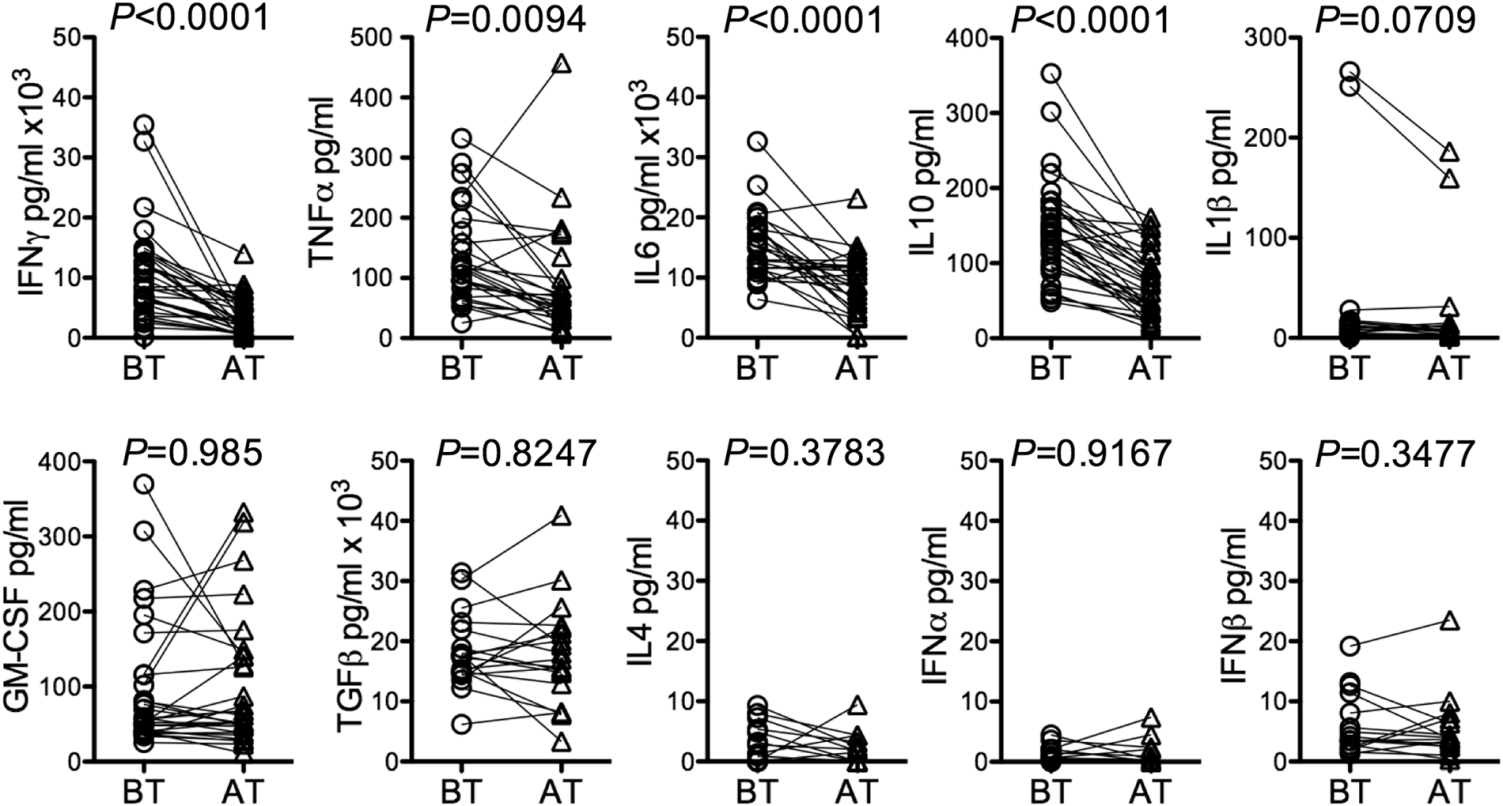
Comparison of cytokine levels in pleural fluids from patients with tuberculous pleuritis before and after antibiotics treatment. The indicated cytokines were assayed by ELISA in pleural fluids of 28 patients. *P* values by paired *t*-test are indicated.

We measured the levels of cytokines IFNγ, TNFα, IL4, IL6, IL10, IL1β, TGFβ, GM-CSF, IFNα and IFNβ in the paired pleural fluids by ELISA. The levels of IFNγ, TNFα, IL6 and IL10 were significantly decreased following antibiotics treatment while the rest did not change significantly (Fig. 2). Intracellular IFNγ, TNFα, IL6 and IL10 were assayed in various cell types after *in vitro* stimulation with PMA plus ionomycin. Consistently, the percentages of CD4^+^ T cells positive for intracellular IFNγ, TNFα, IL6 and IL10 decreased and the percentages of CD8^+^ T cells positive for intracellular IFNγ and TNFα also decreased (Figure S3A, S3B). Similarly, the percentages of non-T cells positive for intracellular TNFα also decreased. RT-qPCR showed that in pleural macrophages the level of IL6 and IFNγ transcripts was significantly decreased following antibiotics treatment (Figure S3C). In the paired samples, change in cytokine levels in pleural fluids was negative correlated with change of macrophage markers (Figure S4). For example, IFNγ, TNFα, IL6 and IL10 level was negatively correlated with the expression of CD80 and CD163, whereas TGFβ and GM-CSF level was negatively correlated with CD206. Taken together, antibiotics treatment reduces cytokine levels in the pleural fluid by modulating functionality of not only T cells but also non-T cells such as macrophages.

### Pleural macrophages polarize to an M2-like phenotype following antibiotics treatment

**Figure 3:**
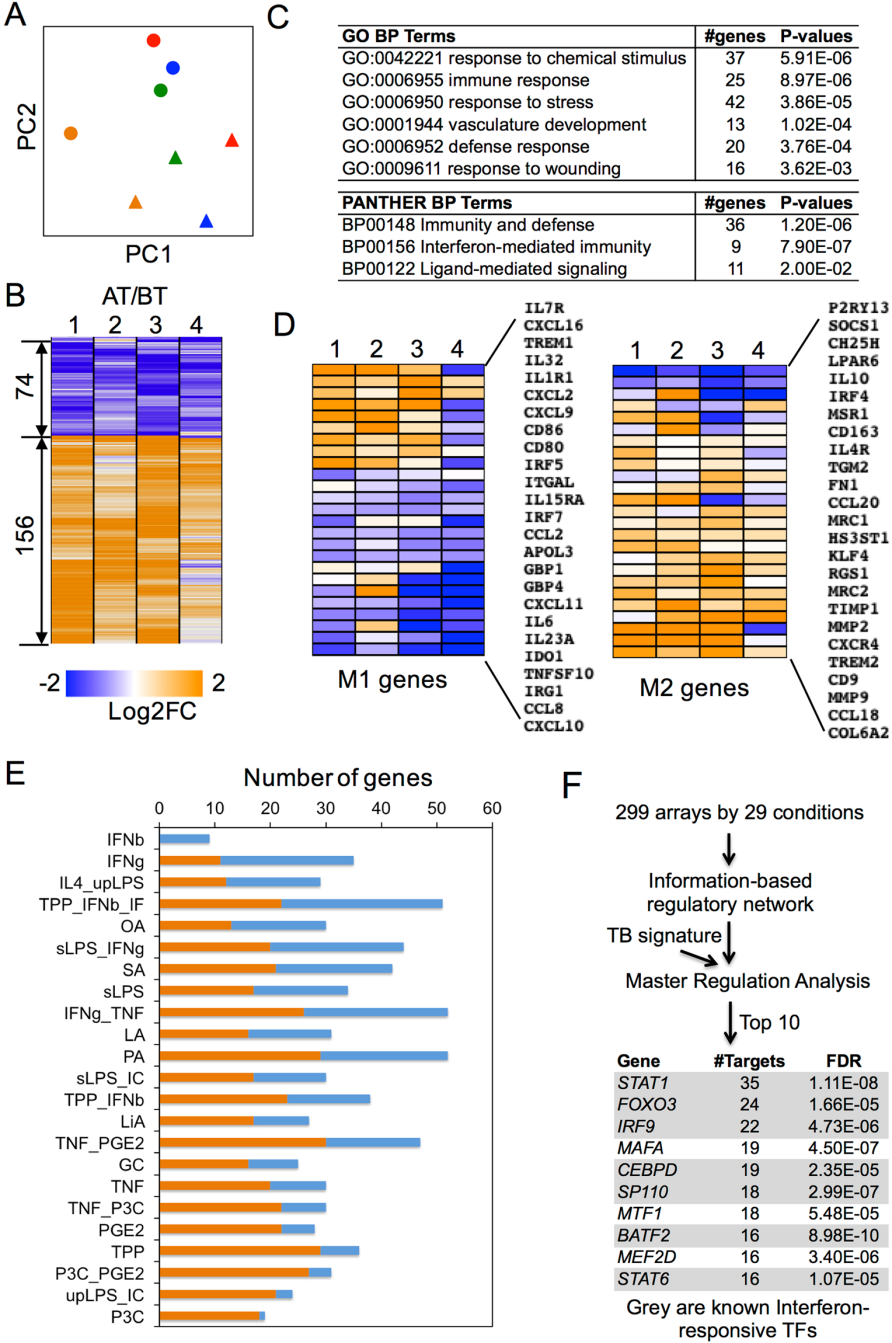
Pleural macrophages are polarized to an M2-like phenotype by antibiotics treatment. (**A**) Principal component analysis of transcriptional profiles of pleural macrophages from four patients (red, orange, blue and green) before (circle) and after (triangle) antibiotics treatment. (**B**) Heatmap showing differentially expressed genes in four pairs of samples. (**C**) List of the most significantly enriched pathways of differentially expressed genes. (**D**) Heatmap of M1 and M2 gene expression in pleural macrophages before and after antibiotics treatment. (**E**) Comparison of differentially expressed genes to gene signatures of hMDMs stimulated by 23 different stimuli (Xue *et al*. 2014). Shown are number of differentially expressed genes in pleural macrophages that are either positively (orange bar) or negatively (blue bar) correlated with the corresponding gene expression in hMDMs following indicated stimulation. (**F**) List of top 10 transcription factors that were identified to regulate the differentially expressed genes.

To better understand the effect of antibiotics on pleural macrophages, we determined the transcription profiles of pleural macrophages before and after HRZE treatment by RNA-Seq of four pairs of pleural macrophage samples with yielding high quality of total RNA. Based on the overall transcription profiles, principle component analysis (PCA) grouped samples before treatment together and samples after treatment together (Fig. 3A), suggesting the sufficient samples to capture the treatment response. Differential expression analysis revealed that 156 genes were up-regulated and 74 genes were down-regulated significantly after antibiotics treatment (Fig. 3B and Table S1). The differentially expressed genes were enriched with immune regulation and stress response pathways (Fig. 3C and Table S2), including response to chemical stimulus (37 genes, *P* = 5.91e-6), immune response (25 genes, *P* = 8.97e-6), response to stress (42 genes, *P* = 3.86e-5), and immunity and defense (36 genes, *P* = 1.2e-6). Regardless of the enriched category, up-regulated genes were enriched with vascular development and extracellular matrix remodeling, while down-regulated genes were enriched with interferon-mediated immunity (Table S2). Moreover, most M1 marker genes, such as *IL6*, *IL23A*, *IDO*, *CCL8* and *CXCL10*/11, were down-regulated whereas M2 marker genes, such as *IL10*, *IRF4*, *CD163*, *IL4R*, *MRC1*/2 and *CCL18*, were up-regulated (Fig. 3D). These results suggest that at the genome level, pleural macrophages are polarized to an M2-like phenotype following HRZE treatment.

Twenty-two of the 74 down-regulated genes were also down-regulated in previous transcriptional analysis of peripheral blood mononuclear cells (PBMCs) in TB patients post antibiotics treatment^22,23^, whereas only 4 of the 156 up-regulated genes were up-regulated in PBMCs. The 22 down-regulated genes were enriched with interferon-mediated immunity (7 genes, P = 1.2e-10) and immunity and defense (10 genes, P = 8.0e-6), including OAS (2′-5′-oligoadenylate synthetase) and IFIT (Interferon-induced protein with tetratricopeptide repeats) family members. We also compared expression profiles in pleural macrophages to the gene expression modules in human monocyte-derived macrophage (hMDMs) stimulated by various stimuli^25^. Most differentially expressed genes in pleural macrophages before and after antibiotics treatment were negatively correlated with IFNγ/TNFα stimulating modules and positively correlated with P3C or TNF_PGE2_P3C (TPP) simulating modules through TLR2 (Fig. 3E). Based on master regulation analysis^26^ of the signature genes and genome-wide regulatory network by Xu *et al*.^25^, 7 of the top 10 identified transcription factors (TFs) were interferon responsive, including STAT1, FOXO3 and IRF9 (Fig. 3F). These results suggest that while IFNγ and TNFα exert significant effect on pleural macrophage polarization, antibiotics may exert separate effect.

### Antibiotics exert direct effect on pleural macrophages

**Figure 4:**
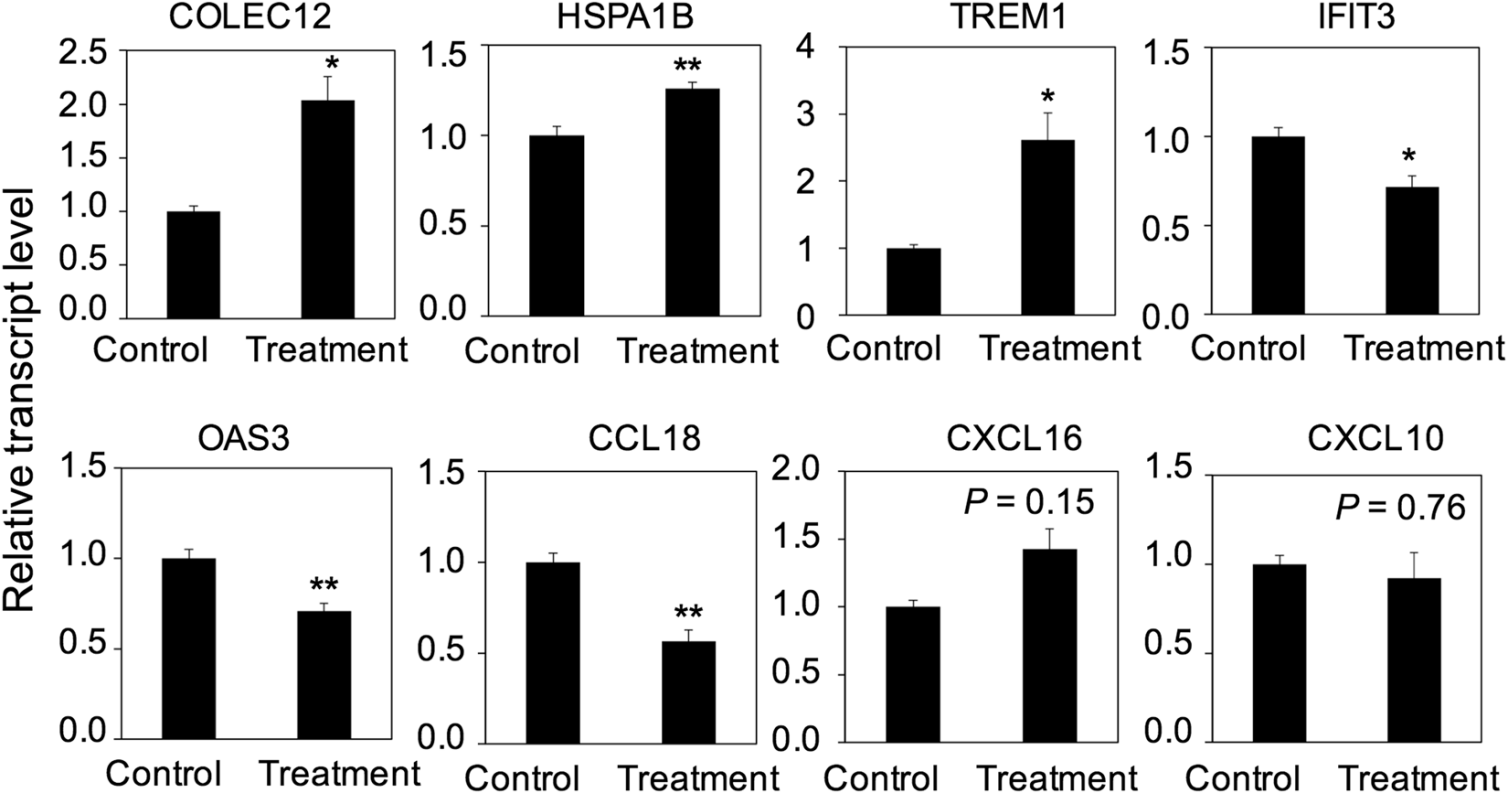
Comparison of gene expression in pleural macrophages with and without antibiotics treatment *in vitro*. Pleural macrophages were isolated from five patients before antibiotics treatment and cultured *in vitro* for 36 hours with or without antibiotics. Cells were collected for RNA isolation and real-time PCR to quantify the transcript level of the indicted genes. Transcript level of each gene was normalized to GAPDH (ct). The ratio of ct with and without antibiotics is shown. Error bar, SEM; *P < 0.05; **P < 0.01.

Gene expression changes in pleural macrophages after antibiotics treatment are enriched in GO term of “response to chemical stimulus” and most of these up-regulated genes were not captured in the whole-blood treatment-specific signature, raising the possibility that antibiotics exert direct effect on macrophage polarization. To test this possibility, we purified pleural macrophages from patients before antibiotics treatment but treated these macrophages with antibiotics *in vitro*. We selected 8 signature genes (*COLEC12*, *HSPA1B*, *TREM1*, *CCL18*, *OAS3*, *IFIT3*, *CXCL16*, *CXCL10*) to monitor the change of gene expression by real-time PCR. These genes not only belonged to “response to chemical stimulus” but to other functional categories such as “immune response” and “response to stress”. Expression of five genes was altered by antibiotics treatment *in vitro*: The levels of *COLEC12*, *HSPA1B* and *TREM1* transcripts were up-regulated, whereas the two IFNγ-mediated antiviral genes *OAS3* and *IFIT3* were down-regulated (Fig. 4), consistent with the *in vivo* results. However, *CCL18*, a typical M2 marker, was down-regulated *in vitro* but up-regulated *in vivo* following antibiotics treatment, and *CXCL16* and *CXCL10*, typical M1 markers and known IFNγ-responsive genes, were not significantly changed *in vitro*. These results show that anti-TB antibiotics directly modulate differentiation status of pleural macrophages.

## Discussion

TPE is one of the most common forms of extrapulmonary tuberculosis. Pleural macrophages exhibit an M1-like phenotype producing pro-inflammatory cytokines and contribute to the initiation and development of TPE^8,27,28^. No study has investigated the treatment-specific response of pleural macrophages in patients with tuberculous pleuritis. Here we examine the phenotypic and functional changes of pleural macrophages post antibiotics treatment by assaying their expression of surface markers, phagocytic function, secretion of cytokines and genome-wide transcriptional profiles. Our results from different analyses show that pleural macrophages are rapidly polarized from M1-like to M2-like phenotype after antibiotics treatment *in vivo*.

Differentiating monocytes or macrophages express higher level of CD14 and HLA-DR than circulating or resident monocytes. The higher expression level of CD14 and HLA-DR in pleural macrophages comparing to monocytes (Supplementary Figure S5A,D), suggests pleural macrophages are not monocytes. However, pleural macrophages before treatment have lower FSC/SSC comparing to pleural macrophages post treatment (Supplementary Figure S5D), suggesting pleural macrophages before treatment tend to be in intermediate status during monocytes to macrophage differentiation. Since the total number or proportion of pleural macrophages did not change before and post antibiotic treatment (Supplementary Figure 1G), additionally the expression levels of CD14+ were slightly elevated post treatment (Supplementary Figure S5B,C) and CD206^low^ pleural macrophages had lower FSC/SSC comparing to CD206^hi^ pleural macrophages (Supplementary Figure S5E), these results suggest that CD206 expression changes are most likely due to the differentiation of pleural macrophages induced by antibiotics treatment but not the reduction of monocytes with low expression of CD206.

Our transcription analysis of pleural macrophages before and after antibiotics treatment identified a treatment-specific signature of 230 genes, which significantly enriched a down-regulated functional pathway of “interferon-mediated immunity”, such as the interferon-related antiviral gene families^29^, IFIT (*IFIT1*, *IFIT2*, *IFIT3*, *IFIT5* and *IFITM1*) and OAS (*OAS1*, *OAS2* and *OAS3*). In previous studies, gene expression analysis of the whole blood of TB patients also showed interferon signaling and innate immune response genes were gradually down-regulated post antibiotics treatment^22,23^, consistent with the decreased level of IFNγ in the patient serum^30^. These results indicate interferon-mediated genes are significantly responsive to antibiotics treatment not only in the peripheral blood but also in the TPE. Moreover, differential expressed genes are negatively correlated with IFNγ and/or TNFα stimulating modules and regulated by interferon responsive TFs, indicating the decrease of IFNγ and TNFα in the TPE exerts significant effect on pleural macrophage polarization post antibiotics treatment. However, a significant fraction of treatment-specific genes was modulated by direct antibiotics treatment of purified pleural macrophages *in vitro*, demonstrating antibiotics may exert independent effects on the pleural macrophage polarization.

Studies have suggested that single anti-TB antibiotics exerts direct immunomodulatory effect on immune cells^15–19^. Among the treatment-specific 230 signature genes, most of them were not present in the whole-blood treatment-specific signature^22,23^ nor IFNγ and/or TNFα stimulating modules, suggesting anti-TB antibiotics might directly regulate gene expression in pleural macrophages. As shown by *in vitro* antibiotics treatment of purified pleural macrophages, the expression of five out of eight selected genes was significantly changed. *COLEC12*, *HSPA1B* and *TREM1* were up-regulated as in *in vivo*. *COLEC12* is a C-lectin scavenger receptor associated with host defense by binding to and removal of microorganisms^31,32^. *HSPA1B* encodes a 70 kDa heat shock protein and has been reported to activate macrophages and enhance phagocytosis^33,34^. *TREM1* plays a crucial role in fine-tuning of inflammatory response by enhancing or inhibiting TLR signals through NFκB and PU.1^35–37^. TREM1-related signaling was identified as the most important activated pathway during active TB disease in blood transcriptome analysis from multiple data sets^38^. Two antiviral genes *IFIT3* and *OAS3*, IL10-inducing and IFNγ-suppressing gene *CCL18* ^39^ were down-regulated, while strong IFNγ-inducing genes *CXCL10* and *CXCL16* ^39^ were not changed. *IFIT3* and *OAS3* can be induced by interferon signal or Pattern Recognition Receptors (PRRs) activation^29^. Interestingly, comparing to expression profiles of human monocyte-derived macrophages (hMDMs) to various stimuli, the most positively correlated stimulus is the TLR2 ligand P3C (Pam3Cys) and combination of LPS and Immune Complex (LPS_IC). Anti-TB antibiotics treatment induced the *TLR2* and *TLR4* expression in lymphocytes and CD14^+^ monocytes in pulmonary tuberculous patients^40^. These results suggest that anti-TB antibiotics might directly modulate the TLR signaling to regulate the gene expression in pleural macrophages. The master regulation analysis also identified several non-interferon or TNF responsive TFs that regulate the differentially expressed genes, such as *MAFA*, *MTF1* and *MEF2D*. These non-interferon or TNF responsive TFs could play a key role in modulating pleural macrophages response to anti-TB antibiotics. Thus, the efficacy of antibiotics in treating tuberculous pleuritis may depend not only on direct killing of mycobacteria but also on modulating the function of pleural macrophages. Consistent with this notion, differentiation of pleural macrophages to M2-like phenotype may promote tissue repair.

Current management of TB treatment lacks robust tools to monitor the treatment efficiency in the early stage during the long-term antibiotics treatment, especially for the treatment of multidrug-resistant TB patients^2–4^. We show that the pro-inflammatory mediators IFNγ, TNFα and IL6 in TPE were decreased significantly post antibiotics treatment as short as 2~3 days. The reduction of these pro-inflammatory cytokines was also observed in the serum, but not until 2~4 months post treatment^30^. Because TPE is more proximal to the site of mycobacterial infection, the more rapid change of pleural cytokines as well as pleural macrophage phenotype could be valuable prognostic biomarkers to monitor the efficiency of antibiotics treatment in TB patients with tuberculous pleuritis.

## Methods

### Patients and study design

A total 171 adult patients with tuberculous pleuritis were enrolled in this study. Participants were recruited from the Infectious Disease Hospital of Changchun, China. Thirty-six patients with serious comorbidities, encapsulated pleural effusion, other lung diseases, HIV co-infection, or contraindications to pleural effusion puncture were excluded from this study. All patients received a full 2 months of combination antibiotics treatment (HRZE) with standard doses of isoniazid (H, 300 mg/d), rifampicin (R, 450 mg/d), pyrazinamide (Z, 1500 mg/d) and ethambutol (E, 750 mg/d) followed by 4-month HR treatment without steroid therapy. Patients underwent thoracentesis (diagnostic and therapeutic) before chemotherapy and/or after 2~10 days of chemotherapy before pleural effusion disappeared. The study protocol was approved by the Ethics Committee of the Infectious Disease Hospital of Changchun and The First Hospital of Jilin University. All experiments were performed in accordance with the relevant guidelines and regulations. In addition, written informed consent was obtained from each subject.

### Sample acquisition

TPE was obtained by thoracocentesis under conscious sedation and local anaesthesia using a 14 G Klatskin needle. 50~150 ml of urokinase-anticoagulated TPE was immediately deposited in 50 ml tube and centrifuged at 1500 rpm at 4 °C for 10 minutes. The cell-free PE was collected and stored at −80 °C before use. Pleural effusion mononuclear cells (PEMCs) were separated immediately on Lymphoprep (Fresenius Kabi Norge AS, Norway) density gradients from cell pellet.

### Cell surface and intracellular cytokine staining

After pre-incubation with human Fc-receptor binding inhibitor (eBioscience, USA) for 15 minutes on ice, fresh PEMCs were stained with fluorochrome-conjugated antibodies of CD14, CD80, CD86, CD163, CD206, CD3, CD4, CD8, CD20 and CD56 (BD Biosciences, USA) at 4 °C in the dark for 20 minutes. Cells were washed, centrifuged and resuspended in cold FACS Buffer (PBS containing 0.1% BSA and 0.01% sodium azide) plus PI before flow cytometry analysis. For intracellular cytokine staining, PEMCs were first plated and stimulated with 50 ng/ml PMA and 1 μM Ionomycin (Sigma-Aldrich) supplement with GolgiStop™ (BD Biosciences) for 4 hours or 18 hours. Cells were stained with Live/Dead^®^ Fixable Dead Cell green fluorescent reactive dye (Invitrogen, USA), then fixed and stained with IFNγ, TNFα, IL6, IL10, IL4 and IL2 using Cytofix/Cytoperm Kit (BD Biosciences). Flow cytometry was performed using a LSR Fortessa cytometer (BD Biosciences) and data were analyzed using Flowjo7.6.1 software.

### ELISA

TPE supernatant were harvested and assayed for IFNα, IFNβ, IFNγ, TNFα, IL-1β, IL-4, IL-6, IL-10, TGFβ, and GM-CSF production by ELISA according to the manufacturers’ protocol from eBioscience, R&D, Abcam, Elabscience and Dakewe Biotech Co.

### Pleural macrophage purification, RNA isolation, RNA-sequencing and data analysis

Pleural macrophages were purified from freshly isolated PEMCs using human CD14 MicroBeads (Miltenyi Biotec, USA). Briefly, cells were passed through a magnetic column as CD14-depleted cells. Labeled cells were collected as purified macrophages. Macrophages were further purified by seeding in 60-mm dish in complete RPMI 1640 medium (Gibco, USA) containing 10% FBS (BI, Israel), 100 U/ml of penicillin and streptomycin. Cells were incubated at 37 °C for 20 minutes and washed off the unattached cells. The purity of macrophages were assessed by Giemsa staining to ensure >90% purity. 0.5~1 × 10^6^ macrophages were collected into 1 ml of TRIzol (Ambion, USA) for RNA extraction.

The quality and quantity of total RNA were assessed with a RNA-6000 Nano LabChip on a 2100 Bioanalyzer (Agilent Inc., USA). cDNA libraries were prepared with SMARTer Universal Low Input RNA Kit (Clontech, USA). The libraries were sequenced by HiSeq. 2000 100 PE (Illumina, USA). Paired sequences were aligned to the human genome (version hg19) using Tophat2^41^. Raw counts of each gene for each sample were calculated by HTseq^42^. Differentially expressed genes between paired samples before and after treatment were performed using edgeR at *P*-value < 0.05 with a cutoff of 2-fold change^43^. Differentially expressed genes were annotated using online functional enrichment analysis tool DAVID^44^. RNA-seq data are available in GEO database under accession code GSE85037.

To compare to the spectrum of human macrophage activation^25^, signature genes of each stimulus were matched to our 230 treatment-specific signature genes based on their expression change trends. Master regulation analysis was performed as previously described^26^. The spectrum data^25^ was used to construct an information-based regulatory network and applied to search for the key transcription factors that regulate the 230 treatment-specific signature genes.

### *In vitro* anti-TB drug treatment

A combined dose of HRZE for *in vitro* treatment was used to mimic the *in vivo* treatment based on the maximum human blood plasma concentrations (Cmax) of approximately 12.4 µg/ml H, 4.0 µg/ml R, 58 µg/ml Z and 4.3 µg/ml E during chemotherapy respectively^45^. Pleural macrophages were purified from PEMCs as described above and incubated with or without HRZE in a 6-well plate at a concentration of 1 × 10^6^ cells/well for 36 hours.

### Real time RT-PCR

Pleural macrophages from *in vitro* treatment were collected to isolate total RNA using TRIzol. cDNA was reverse-transcribed using Maxima FirstStrand cDNA Synthesis Kit (Thermo, USA) according to the manufacturer’s instructions. RT-qPCR was performed in ABI Plus one (Applied Biosystems, USA) using FastStart Universal SYBR Green Master (Roche, Germany). Either the 18S ribosomal RNA (forward: GCGGCTTTGGTGACTCTA, reverse: CTGCCTTCCTTGGATGTG) or *GAPDH* (forward: CGGATTTGGTCGTATTGGG, reverse: CGGATTTGGTCGTATTGGG) was used as an internal control for normalization. The gene-specific primer sets were purchased from Qiagen as following: *COLEC12* (Cat. QT00080192), *HSPA1B* (Cat. QT01668212), *CCL18* (Cat. QT00024066), *CXCL10* (Cat. QT01003065), *IFIT3* (Cat. QT00100030) and *OAS3* (Cat. QT01005277) or synthesized as *CXCL16* (forward and reverse primers: AAACCACCATTCACACTGCG and AGCCACAGTTTACCCTCACAA), *TREM1* (AAGCTCCACCCAAGTCAACTG, and CATCCTCTCAGCACACAGACT), *IL1β* (CAGAAGTACCTGAGCTCGCC, and AGATTCGTAGCTGGATGCCG), *IL6* (GAACTCCTTCTCCACAAGCG and GAAGAGGTGAGTGGCTGTCTG), *IL10* (GGGAGAACCTGAAGACCCTCA and TGCTCTTGTTTTCACAGGGAAG), *IFNγ* (CATCCAAGTGATGGCTGAACTG and TCGAAACAGCATCTGACTCCTTT), *TNFα* (ATCCTGGGGGACCCAATGTA, and AAAAGAAGGCACAGAGGCCA), *TGFβ* (CTGTATTTAAGGACACCCGTGC, and ATGACACAGAGATCCGCAGTC), *GM-CSF* (CAGCCCTGGGAGCATGTG and CATCTCAGCAGCAGTGTCTCTAC).

### Statistical method

in the method as following “Data for flow cytometry and qPCR were presented as mean ± SEM. Statistical analysis was performed with Prism5 (GraphPad Software, San Diego, CA). Statistical significance between before and after treatment groups was defined as *P* < 0.05, and obtained with a paired or unpaired two-tailed t-test for paired and unpaired samples, respectively. Differences are noted as *P < 0.05, **P < 0.01.

## Electronic supplementary material

Supplementary figures and tables
