# Supplementary material for: Antibiotics induce polarization of pleural macrophages to M2-like phenotype in patients with tuberculous pleuritis
Source: Sci Rep. 2017 Nov 3;7:14982. doi: 10.1038/s41598-017-14808-9 (PMC5670217; doi:10.1038/s41598-017-14808-9)
Supplement: Supplementary file 1 — Supplementary figures and tables [file 41598_2017_14808_MOESM1_ESM.pdf]

Supplemental information

Antibiotics induce polarization of pleural macrophages to M2-like phenotype in patients with tuberculous pleuritis

Sisi Wang[1,#], Jian Zhang[1,2,#], Liyan Sui[1], Hao Xu[1], Qianling Piao[1], Ying Liu[1,3], Xinglong Qu[1,3], Ying Sun[3], Lei Song[3], Dan Li[3], Liping Peng[3], Shucheng Hua[1,3*], Guangan Hu[4,*] , Jianzhu Chen[1,4,*]

1. Department of Translational Medicine, The First Hospital, Jilin University, Changchun, 130061, China.
2. The Infectious Disease Hospital of Changchun, Changchun, Jilin, Changchun, 130123.
3. Department of Respiratory Medicine, The First Hospital, Jilin University, Changchun, 130021, China.
4. Koch Institute for Integrative Cancer Research and Department of Biology, Massachusetts Institute of Technology, 77 Massachusetts Avenue, Cambridge, MA, USA 02139.

# These authors contribute equally.
* corresponding authors: Guangan Hu (gahu@mit.edu) , or Jianzhu Chen (jchen@mit.edu), or Shucheng Hua (shuchenghua@eyou.com)

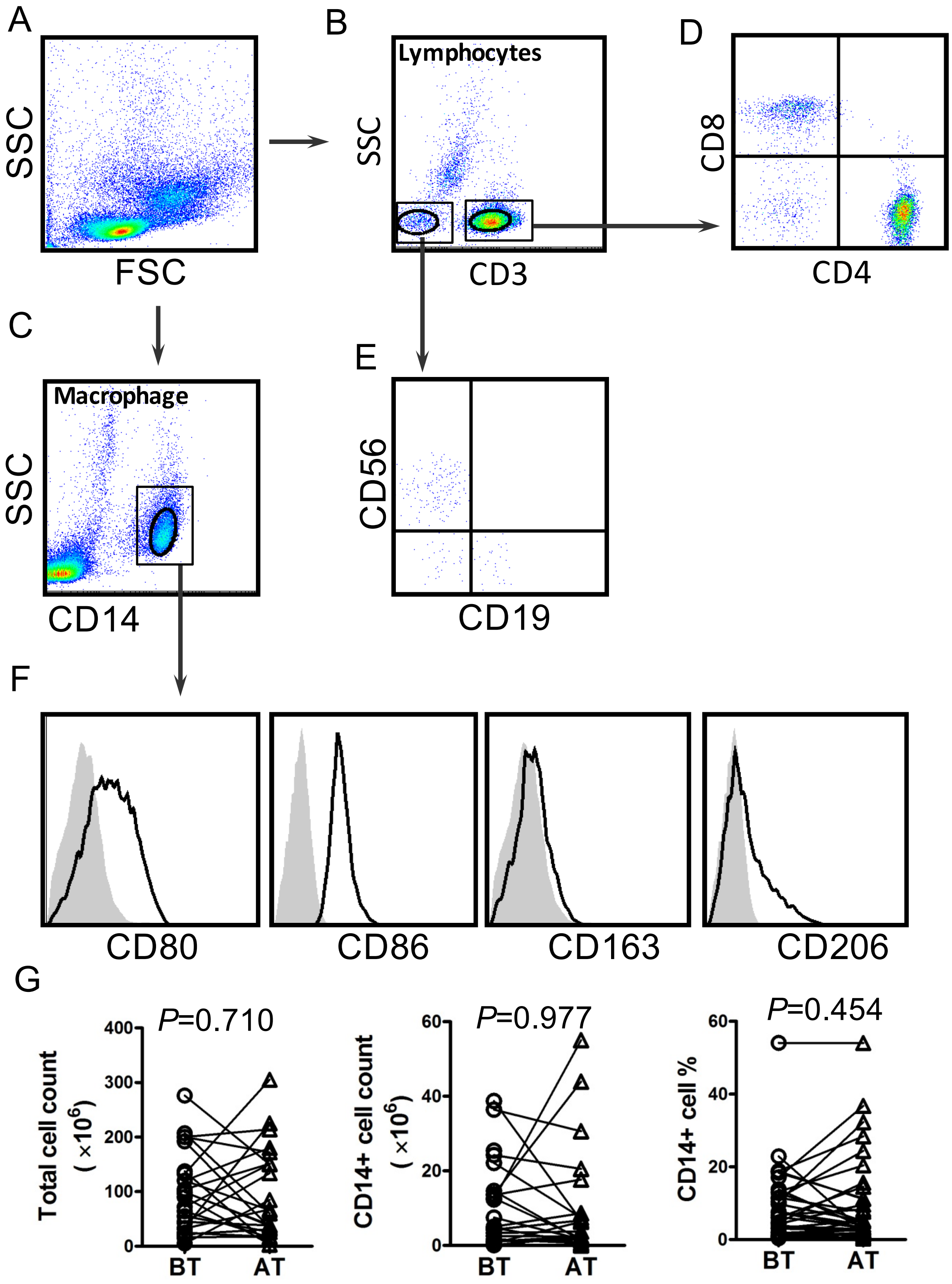

Figure S1. Flow cytometry analysis of various immune cells in tuberculous pleural fluids. Cells were stained for various markers and analyzed by flow cytometry. Representative staining profiles showing (A) forward scatter (FSC) vs. side scatter (SSC), (B) CD3 vs. SSC, and (C) CD14 vs. SSC gating on live cells; (D) CD4 vs. CD8 on $CD3^{+}$ live cells; (E) CD19 vs. CD56 gating on $CD3^{-}$ live cells. (F) Histogram showing CD80, CD86, CD163, and CD206 gating on $CD14^{+}$ cells of staining with isotype (shaded trace) and each antibody (black trace). (G) toal number of pleural cells, total number and proportion of pleural macrophages before and after antibiotic treatment in TPE. *P* values by paired *t*-test between before treatment (BT) and after treatment (AT) are indicated.

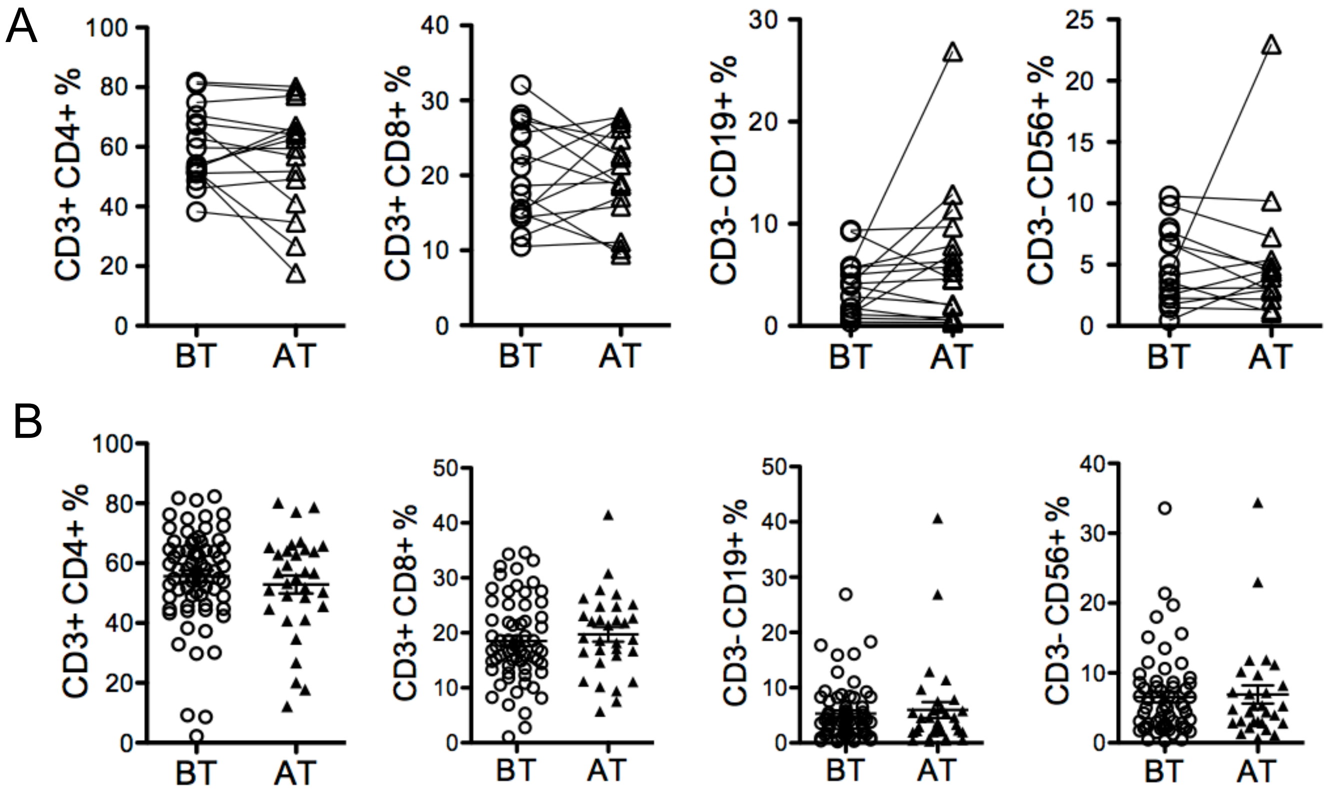

Figure S2. Comparison of percentages of $CD4^{+}$ and $CD8^{+}$ T cells, $CD19^{+}$ B cells and $CD56^{+}$ NK cells before (BT) and after (AT) antibiotics treatment. (A) Paired samples (n=29). (B) Unpaired samples (n=128).

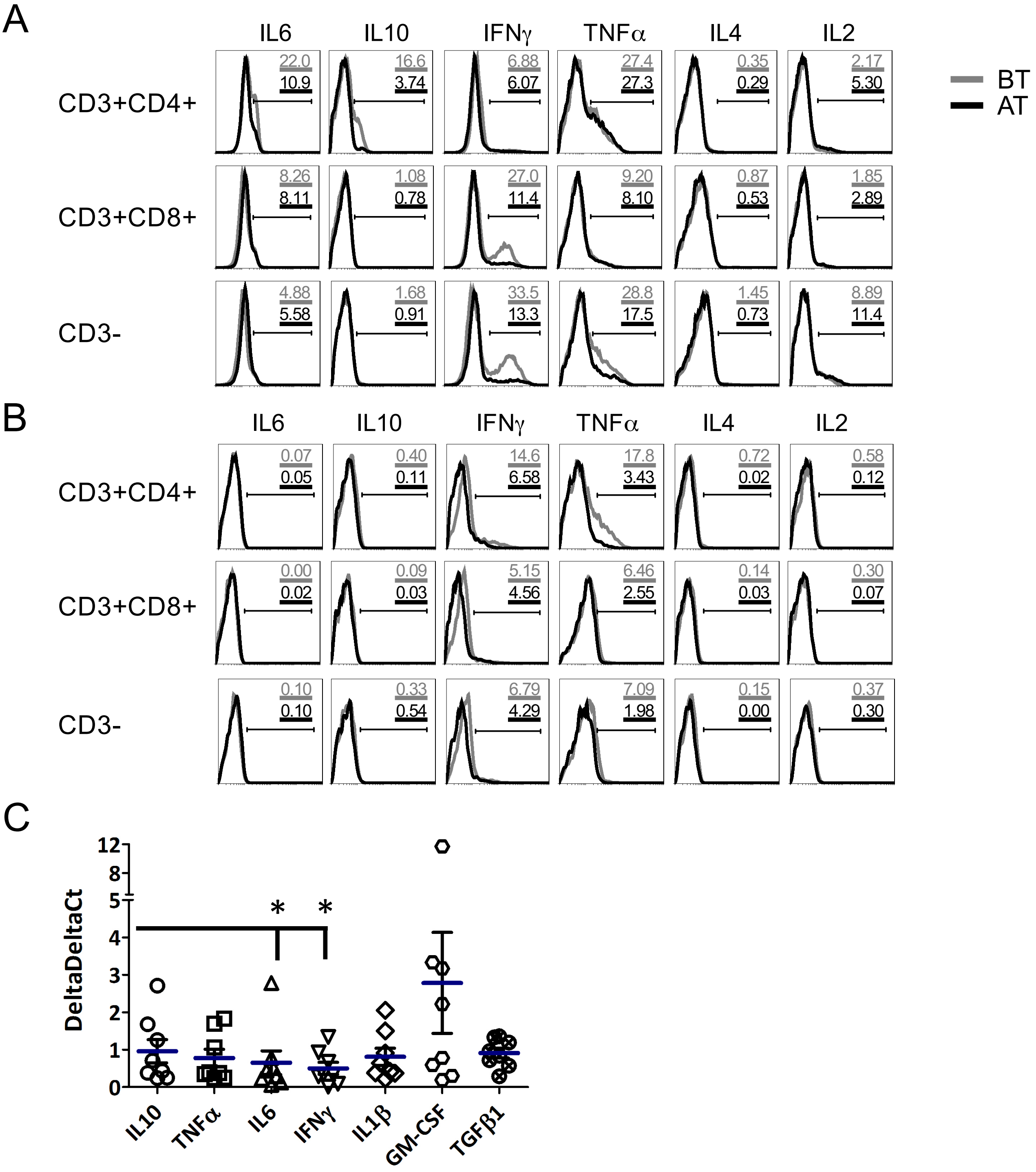

Figure S3. A-B, Intracellular staining of cytokines in pleural leukocytes before and after antibiotics treatment. Leukocytes were isolated from pleural fluids and stimulated *in vitro* for 18 hours (A) and 4 hours (B) with PMA and ionomycin in the presence of GolgiStop. Representative histograms show intracellular cytokine staining gating on $CD3^{+}CD4^{+}$, $CD3^{+}CD8^{+}$, and $CD3^{-}$ cells (n=6). AT, black trace and BT, grey trace. C, Comparison of the transcript levels of indicated cytokine genes in pleural macrophages isolated from patients before and after antibiotics treatment. Transcript level of each gene was normalized to GAPDH (ct). The ratio of ct before and after treatment is shown (n=8). Error bar, SEM; *, $P<0.05$.

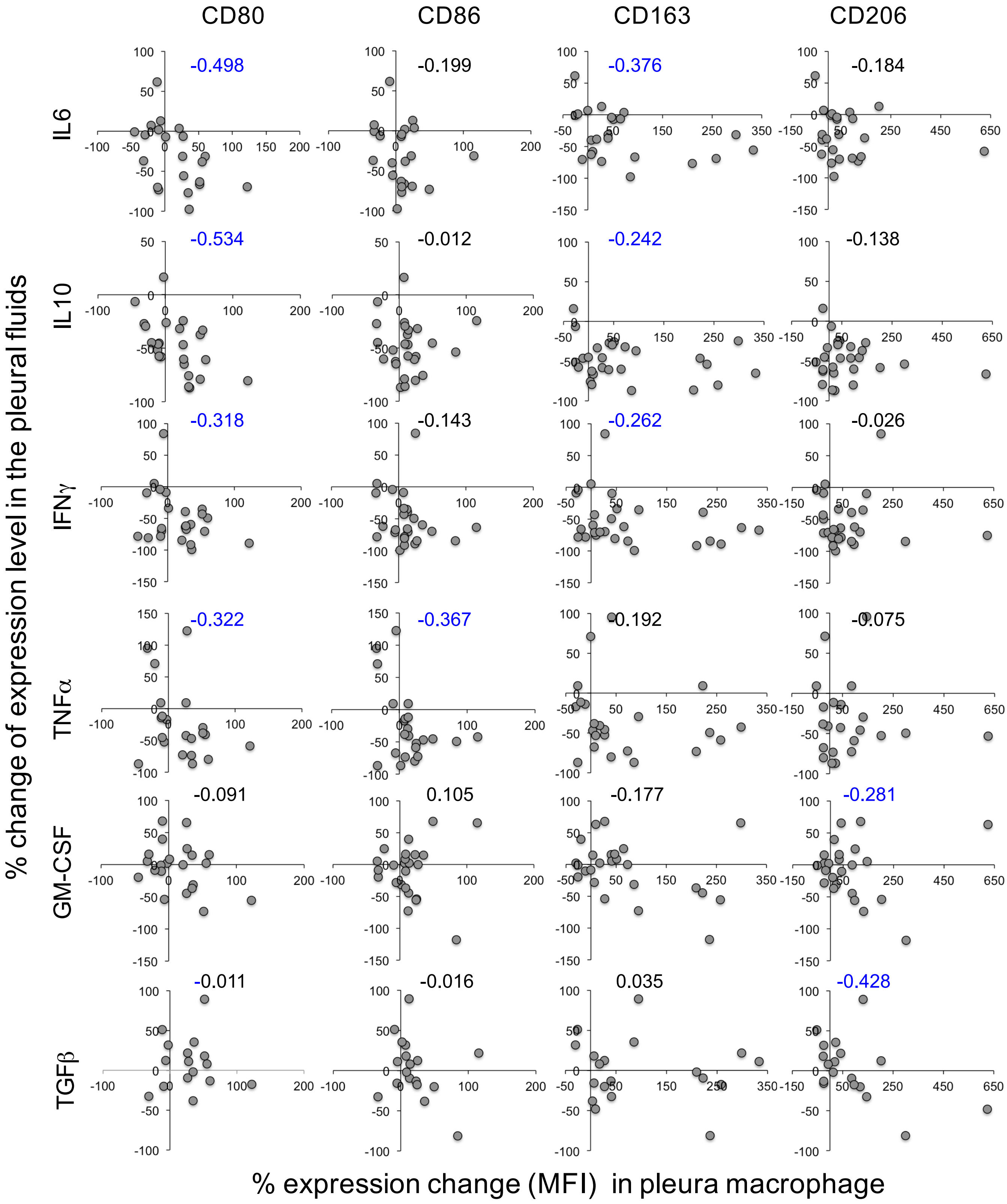

Figure S4. Correlation analysis of changes in cytokine levels in pleural fluids and changes in surface marker expression on pleural macrophages in 29 paired samples before and after antibiotics treatment. The numbers show correlation efficiency.

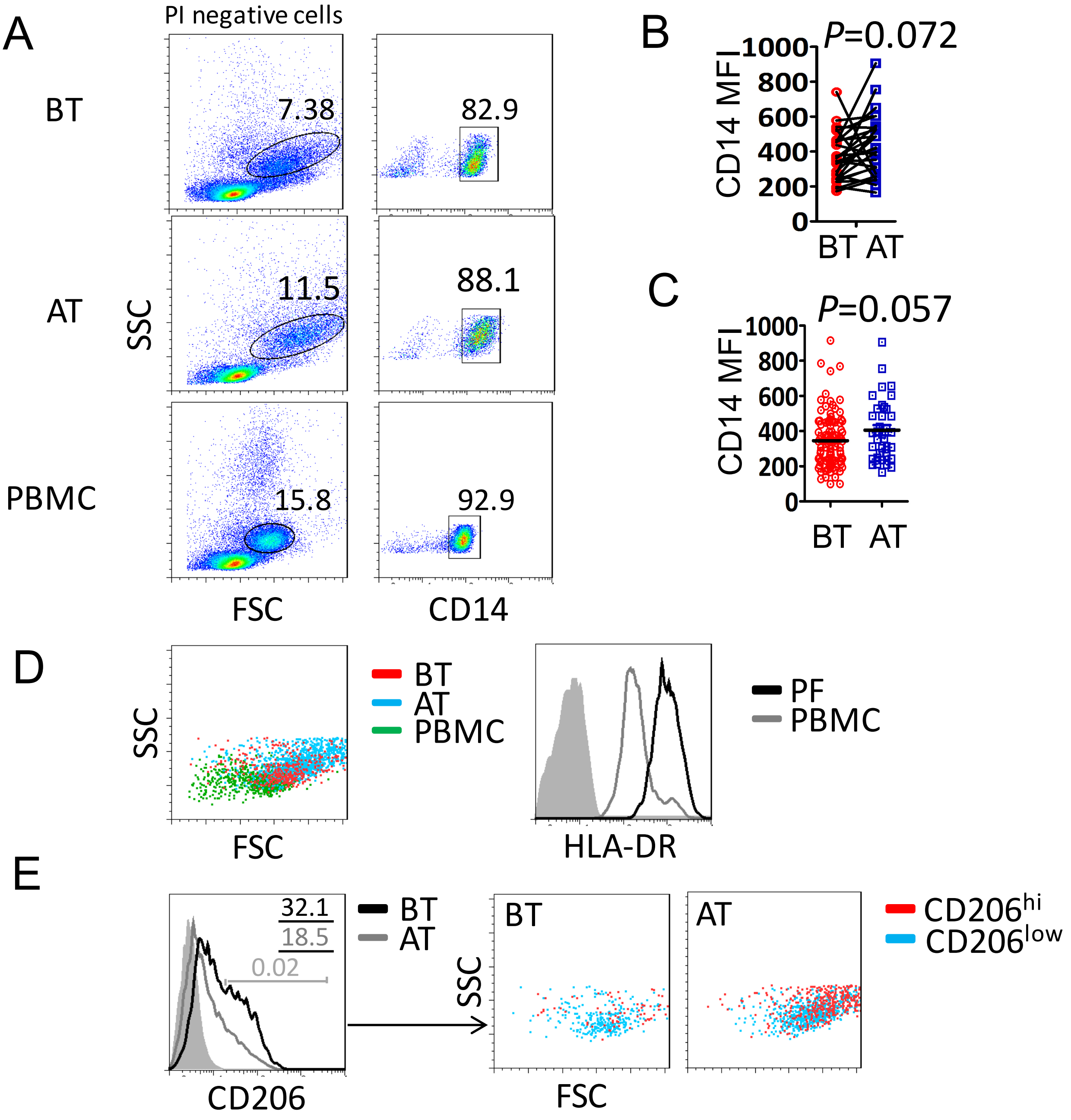

Figure S5. A, The expression of CD14 of monocytes of peripheral blood and pleural macrophages before (BT) and after antibiotics treatment (AT). B-C, Aggregated data from paired samples (B, n=29) and unpaired samples (C, n=128) showing mean fluorescence intensity (MFI) of CD14. *P* values by paired *t*-test are shown between BT and AT. D, The FSC/SSC plotting of monocytes of peripheral blood and pleural macrophages ($CD14^+$ cells) before and after antibiotics treatment. E, The FSC/SSC plotting of $CD206^{hi}$ and $CD206^{low}$ pleural macrophages before and after antibiotics treatment. .

Table S1. The list of 230 treatment-specific signature genes.

Gene	logFC	logCPM	LR	PValue	PF118	PF119	PF128	PF131	PF149	PF151	PF154	PF155
A2M	2.41	8.34	8.91	0.0028373	87.22	747.30	36.16	237.77	216.58	1037.06	59.32	175.71
ABCC3	1.02	3.56	5.38	0.0203772	5.56	10.19	12.55	20.50	4.09	15.25	10.14	15.73
ACP5	1.20	5.48	5.97	0.0145587	23.39	42.70	67.44	79.75	16.07	43.45	14.44	68.60
ADAM8	1.63	6.52	8.38	0.0037943	13.25	88.93	52.95	101.73	39.26	146.75	98.59	192.97
ADAMDEC1	1.75	6.41	4.29	0.0383258	21.62	269.05	6.48	24.70	49.84	182.86	78.16	50.40
ADSSL1	1.64	2.34	4.79	0.0286773	1.59	14.55	1.74	4.18	2.74	12.65	1.89	1.69
AFMID	1.22	2.49	5.22	0.0223881	4.57	12.01	2.84	3.14	3.43	10.51	2.07	6.56
AMY2B	1.03	2.31	4.95	0.0261092	2.85	8.98	2.04	3.39	4.45	7.42	3.53	7.12
ANPEP	1.18	8.50	3.95	0.0468163	328.74	412.28	426.76	554.21	29.08	234.42	280.25	635.17
APOC1	1.93	6.78	5.90	0.0151784	58.97	320.32	16.41	61.11	54.62	311.54	19.51	34.44
APOE	2.81	9.31	7.22	0.0072183	191.34	2190.47	32.80	291.99	173.75	1936.91	87.47	157.69
ARL4C	2.21	6.68	6.71	0.0095784	60.11	291.60	3.19	20.50	21.83	146.10	88.96	189.40
ARRDC2	1.06	5.72	7.71	0.0054891	30.86	83.32	43.23	55.83	22.75	58.88	41.12	85.17
ARRDC3	1.38	7.54	5.80	0.0160139	102.16	351.80	42.58	108.04	101.61	464.44	150.63	167.19
ATG16L2	1.13	4.69	7.13	0.0075773	14.44	56.11	20.93	28.96	12.98	24.46	15.13	34.42
ATP13A3	1.22	7.03	5.24	0.022032	26.58	163.04	55.31	148.61	139.14	292.73	121.77	101.09
AVPI1	1.23	3.47	7.11	0.0076759	3.57	8.94	7.95	17.71	7.93	20.98	6.94	14.09
BAIAP2	1.26	3.40	8.38	0.0038007	5.63	11.06	7.21	17.63	6.49	18.13	5.23	12.67
BAIAP2-AS1	1.59	2.33	8.58	0.0034012	2.46	6.52	2.87	4.61	2.74	8.80	1.70	10.30
BBS1	1.25	1.71	5.28	0.0215613	1.47	7.50	0.93	2.32	3.00	5.06	2.40	3.70
BHLHE41	1.02	6.57	4.78	0.0287524	38.48	167.81	72.76	199.07	54.24	97.59	75.69	56.98
C17orf96	1.80	2.21	8.87	0.0028953	3.17	9.96	1.06	5.36	1.64	9.37	2.59	4.17
C5AR1	1.14	8.51	6.15	0.0131364	179.65	724.94	144.39	303.96	275.27	604.73	301.89	380.40
CCL18	2.25	3.71	9.68	0.0018677	2.19	28.87	3.50	24.78	4.65	22.59	9.56	9.04
CD300LB	1.25	5.32	7.64	0.0057016	18.61	28.34	21.24	73.75	18.52	51.61	33.84	73.93
CD9	1.52	5.48	3.94	0.0470149	9.70	9.02	53.38	146.27	8.93	43.80	14.45	71.57
COLEC12	1.85	3.63	4.60	0.0320567	1.88	12.54	2.05	7.85	5.93	51.98	10.91	5.73
COLQ	1.34	2.94	5.26	0.0218624	4.96	17.96	1.99	4.53	8.47	14.90	2.29	6.55
CREM	1.52	5.39	5.71	0.0169077	11.98	89.50	10.77	18.75	23.20	104.99	35.59	40.78
CSNK1E	1.10	3.07	6.42	0.0113059	4.90	8.03	3.07	9.91	6.85	12.84	6.79	14.70
CST7	1.14	1.55	5.16	0.0231473	1.34	2.39	1.08	3.48	2.37	3.59	2.40	6.45
CXCL16	1.20	8.29	7.53	0.0060581	156.17	388.03	290.46	519.02	123.06	496.48	208.43	323.02
CXXC5	1.45	3.94	9.33	0.0022519	8.65	15.95	9.44	24.73	5.00	27.38	9.93	21.12
CYBRD1	1.44	3.58	6.35	0.0117417	8.68	18.19	3.35	6.77	5.14	31.72	6.86	14.67
CYP27A1	1.65	5.88	6.57	0.0103561	16.74	91.69	7.92	41.56	37.61	142.69	75.72	56.64
DEXI	1.20	4.01	6.19	0.012816	7.09	26.11	24.19	17.41	6.95	20.64	6.41	20.50
DNMBP	1.85	4.51	7.38	0.0065791	5.12	20.35	7.93	17.06	10.54	85.92	10.12	25.12
DPEP2	1.46	3.60	7.15	0.0075031	6.07	32.05	7.43	12.95	4.61	19.19	6.00	9.03
DUSP4	1.19	5.11	4.16	0.0414694	32.11	88.51	3.90	19.42	19.89	31.74	35.62	45.36
EDNRB	1.07	3.93	4.14	0.0418695	5.57	17.96	7.30	6.48	12.42	35.34	11.21	25.63
ENO2	1.85	3.48	6.72	0.0095559	1.56	10.42	5.38	22.22	3.59	27.28	11.65	6.90
EPAS1	1.58	3.98	5.53	0.0186529	4.89	21.75	5.57	11.38	7.52	56.80	8.30	9.70
FBXL20	1.03	2.85	4.59	0.0320746	4.30	14.81	5.42	4.18	4.98	11.13	3.48	9.62
FOXO1	1.61	3.47	9.45	0.0021133	3.51	18.15	3.62	15.67	6.62	19.69	9.49	11.99
GABARAPL	1.13	7.35	7.40	0.0065228	104.27	279.09	80.36	138.55	82.87	204.04	137.04	275.71
GGTA1P	1.55	5.59	5.30	0.0212838	55.32	140.00	4.34	19.85	32.97	78.03	14.83	40.67
GK5	1.04	3.46	4.72	0.0298879	4.72	6.82	9.16	11.21	7.08	24.36	6.14	18.03
GLUD1P3	1.10	1.86	5.20	0.0226073	1.91	7.09	1.58	3.83	1.95	4.98	4.12	3.67
GOLGA8H	1.31	1.38	6.52	0.010643	1.06	2.58	1.24	2.69	1.26	5.29	2.37	4.07
GOLGA8N	1.36	2.06	6.39	0.0114746	1.59	9.43	1.40	3.27	3.25	5.06	3.05	6.42
GPNMB	2.06	8.59	6.96	0.00832	77.89	720.09	80.82	340.57	213.91	1331.67	147.54	165.85
GPRIN3	1.32	5.41	8.46	0.0036333	14.18	44.48	34.22	43.09	22.80	88.46	26.54	66.75
GPX3	3.39	4.45	15.14	9.963E-05	3.45	50.39	3.49	20.30	5.10	52.36	2.73	37.73
HBEGF	1.17	7.75	3.91	0.0479099	102.97	656.85	64.55	107.07	100.17	189.46	213.23	290.62
HDGFRP3	1.51	2.06	7.85	0.0050803	1.41	5.04	3.23	9.93	1.44	4.66	2.59	4.86
HGSNAT	1.13	5.87	7.68	0.0055752	33.56	80.36	32.64	45.22	38.91	113.61	36.83	87.54
HLA-DOA	1.37	7.40	7.22	0.0071983	121.53	243.25	76.08	139.37	120.96	427.23	50.16	171.10
HSD3B7	1.03	5.36	4.58	0.032318	16.90	41.26	39.63	67.64	21.09	94.77	24.47	22.98

HSPA1A	1.29	7.90	4.98	0.0256933	245.29	510.52	123.07	461.79	153.22	267.51	40.36	104.72
HSPA1B	1.66	6.19	6.17	0.0129768	28.36	276.10	16.78	44.70	32.41	77.51	39.57	70.02
ICOSLG	1.16	5.52	9.03	0.00265	23.79	70.29	39.61	53.29	24.10	69.65	27.52	58.97
IFITM10	1.19	4.11	5.66	0.017309	9.02	16.79	11.95	27.14	5.59	39.83	14.05	13.56
IL32	1.23	3.70	3.88	0.04881	8.35	30.58	5.48	15.09	4.19	24.46	11.66	4.41
ITGB5	1.31	4.60	6.95	0.0083868	18.67	33.95	6.00	24.48	20.95	57.49	11.44	21.62
KIAA0753	1.01	1.94	4.68	0.0305964	2.28	7.12	3.28	5.43	3.35	5.11	1.36	2.85
LGMN	1.32	8.18	5.29	0.0214195	213.03	436.83	54.40	278.99	153.73	716.74	271.26	197.83
LILRB5	1.11	3.31	4.49	0.0340801	4.16	5.00	8.40	22.29	6.12	19.14	4.37	9.38
LMNA	1.40	8.25	6.33	0.0118743	114.85	375.37	228.73	879.02	95.66	312.38	202.17	228.35
MKI67	1.61	2.99	4.38	0.0363408	2.55	9.59	1.00	5.46	15.19	24.51	1.34	3.60
MMP14	1.17	6.20	7.11	0.0076604	32.67	130.72	24.61	69.06	57.68	116.14	73.92	83.22
MMP19	1.43	7.68	5.14	0.0233469	59.52	307.47	33.97	167.31	171.42	492.44	258.59	151.62
MMP9	2.10	6.41	5.61	0.0178226	17.78	123.07	3.65	83.95	45.88	175.23	173.32	55.51
MOB3B	1.84	3.73	5.98	0.014496	2.73	5.76	9.90	18.76	2.14	43.93	5.96	16.29
MTSS1	1.40	4.94	6.95	0.0083896	15.30	62.21	7.00	26.85	14.27	49.38	39.06	32.16
NDRG1	1.09	6.60	8.46	0.0036208	47.35	105.33	69.94	163.68	60.61	139.64	70.42	120.65
NEDD9	1.20	4.34	7.01	0.0080841	9.08	25.88	5.80	18.58	21.09	31.99	16.47	32.93
NLRP1	1.14	4.83	7.79	0.0052476	10.75	43.57	19.85	31.00	15.29	33.91	26.82	45.61
NUAK2	1.00	2.49	4.17	0.0412271	2.06	7.84	5.95	4.18	2.28	8.80	5.48	8.36
PAPSS2	1.27	5.11	5.89	0.0151965	10.40	26.52	33.99	50.73	18.24	90.18	16.23	29.41
PC	1.53	2.84	8.93	0.0027992	3.08	7.27	3.72	13.08	5.86	12.92	2.34	8.97
PCYOX1	1.01	3.10	4.63	0.0314391	5.98	16.18	3.91	5.75	8.41	16.30	3.95	8.41
PDE3B	1.10	2.37	4.52	0.0335114	1.56	3.49	3.23	4.60	3.03	8.83	4.98	11.37
PDE4A	1.40	5.91	9.24	0.0023664	19.46	96.73	34.24	60.69	29.95	108.61	52.77	80.10
PHLPP1	1.14	1.80	5.41	0.0200142	1.52	3.71	4.43	6.05	1.38	5.04	1.90	3.69
PIK3IP1	1.25	3.30	4.25	0.0393031	5.39	26.26	6.81	7.07	3.03	19.30	5.85	5.83
PLD2	1.22	4.86	7.79	0.0052641	11.70	53.23	21.95	36.39	14.00	45.74	22.48	26.58
PMP22	1.38	3.78	5.31	0.0211819	5.48	20.65	2.07	6.10	11.00	32.61	13.32	18.47
PPARD	1.07	6.43	6.48	0.0108956	29.51	111.51	54.47	92.99	63.51	174.04	78.63	85.44
PROS1	1.24	2.33	4.84	0.0278714	2.20	4.77	1.93	3.34	3.82	15.13	2.87	6.02
PRUNE	1.13	2.11	5.48	0.0192015	3.58	5.91	3.88	5.30	2.83	7.93	1.09	4.05
RGCC	1.42	7.29	10.28	0.0013463	80.79	234.92	73.98	255.32	69.73	233.65	120.60	180.89
RHBDD2	1.04	4.66	7.06	0.0078964	15.42	28.04	13.78	27.97	11.86	32.79	25.95	45.81
RNASE4	1.72	2.33	7.37	0.0066422	2.08	5.23	0.97	3.58	1.65	8.43	5.14	12.90
RPS6KA2	1.27	2.39	5.79	0.0160836	1.76	4.09	1.88	5.51	5.03	7.59	3.67	11.95
SATB1	2.20	6.05	10.68	0.0010825	16.61	71.69	17.58	54.52	17.17	188.97	39.37	123.93
SDC2	2.75	4.74	8.85	0.0029277	3.25	7.35	8.17	51.18	3.04	86.80	8.13	45.95
SDHAP1	1.03	2.43	4.89	0.0269922	2.29	3.49	4.67	7.89	3.72	9.68	3.12	8.02
SDS	3.05	5.94	12.42	0.0004254	12.04	83.13	8.68	47.34	8.49	215.75	18.03	96.30
SEL1L3	1.37	4.50	8.30	0.0039699	6.51	20.50	22.57	35.79	8.54	33.34	16.07	37.12
SERINC5	1.59	4.78	7.79	0.0052633	7.18	24.59	26.54	43.93	9.20	68.50	12.97	26.04
SERPINE1	1.45	4.91	7.44	0.0063772	11.48	38.87	7.68	38.00	19.67	69.12	29.75	25.34
SERPINF1	1.40	3.79	4.02	0.0450444	4.72	15.69	1.92	4.71	4.67	12.78	20.55	45.15
SERTAD3	1.10	2.81	6.18	0.0129115	3.76	6.25	7.03	10.43	4.21	10.92	3.11	10.15
SH3BP5	1.47	4.26	6.65	0.0099153	3.64	19.74	16.56	26.83	8.92	48.73	13.09	15.85
SH3D21	1.16	2.61	4.30	0.0380446	1.09	7.81	4.24	10.81	6.30	10.66	4.21	3.63
SH3PXD2B	1.28	4.98	7.26	0.0070433	13.46	27.05	35.06	39.08	13.79	64.78	13.80	44.99
SHB	1.46	2.71	7.98	0.0047303	1.67	8.75	3.04	7.77	3.69	11.68	6.62	8.97
SIK1	1.09	7.35	5.41	0.0200518	78.53	352.45	86.39	89.93	112.74	235.98	111.77	237.24
SIPA1L1	1.02	6.99	4.35	0.0369787	39.83	65.62	134.00	179.16	55.47	158.81	104.23	278.71
SLC15A2	1.04	1.96	4.42	0.0356006	3.69	5.57	4.82	4.01	1.16	5.48	1.55	4.78
SLC25A29	1.66	2.80	8.51	0.0035352	2.99	14.89	4.18	11.48	2.87	12.15	2.72	4.61
SLC2A1	1.33	2.98	8.04	0.0045668	2.78	11.94	3.93	8.34	5.37	10.17	6.17	14.47
SLC2A3	1.27	9.01	7.44	0.0063856	215.55	1116.23	296.31	519.22	324.63	727.30	341.14	586.27
SLC40A1	1.59	2.68	4.37	0.0365004	3.84	19.97	1.14	2.29	3.85	16.02	1.66	3.08
SLC6A8	1.01	3.31	4.37	0.0365939	5.86	15.19	6.35	9.19	6.07	24.23	6.03	6.58
SLCO4A1	1.12	2.49	4.82	0.0281686	1.70	7.43	2.29	9.11	4.29	7.47	7.35	5.19
SMOX	1.55	4.42	8.34	0.003877	9.64	28.27	6.50	15.49	10.99	54.52	14.50	31.13
SNX33	1.16	3.27	4.35	0.0370465	2.14	17.92	4.21	6.77	10.20	19.92	7.63	8.36

SOCS6	1.32	3.76	5.92	0.0150088	4.78	10.08	9.96	11.40	6.71	36.23	7.26	21.35
SPP1	2.04	9.70	4.29	0.038253	65.76	210.82	300.38	848.09	401.65	4423.37	93.97	287.29
SPRY2	1.82	3.88	9.89	0.0016589	5.40	29.63	8.37	18.50	3.10	26.21	10.53	16.14
SSBP2	1.14	2.42	5.70	0.0169835	3.73	12.12	2.35	4.81	4.21	7.74	2.76	5.34
ST6GALNA(	1.00	3.24	5.70	0.0169476	7.13	7.81	6.09	14.59	7.54	16.20	4.16	11.76
STAT4	1.49	4.11	5.20	0.0226181	2.81	8.83	9.28	21.29	10.60	60.24	9.58	14.71
SULF2	1.08	7.19	5.85	0.0156036	57.55	122.69	145.69	255.93	47.43	188.64	147.93	200.24
SYTL3	1.32	3.99	6.06	0.013846	5.75	25.42	5.42	9.62	11.63	39.66	12.08	17.66
TARSL2	1.39	2.44	7.86	0.005045	1.73	6.44	4.67	7.62	4.01	7.71	2.19	8.89
TBC1D30	1.16	2.44	4.05	0.0441834	1.96	8.90	3.41	4.08	2.41	13.81	4.93	3.95
TFDP2	1.21	2.73	4.47	0.0344903	2.09	11.82	1.74	3.17	7.06	13.05	5.55	8.73
TFRC	1.21	7.96	3.88	0.0487552	87.77	517.57	84.18	107.07	132.85	589.88	255.81	215.93
THBD	1.46	7.18	10.00	0.0015666	85.48	206.19	89.17	143.30	55.36	237.11	77.86	267.60
THBS1	1.83	9.73	5.46	0.0194654	215.16	664.13	41.40	287.48	722.59	2364.18	756.60	1736.32
THBS3	1.00	3.52	6.16	0.01304	6.16	20.46	7.18	10.31	9.15	17.49	7.62	13.65
TMEM2	1.02	6.65	6.73	0.0094727	49.67	127.99	55.31	89.14	71.51	195.56	85.76	127.05
TMEM91	1.02	1.80	4.18	0.0410194	2.90	8.94	1.98	2.57	2.01	4.84	1.81	3.19
TNFRSF12A	1.46	1.75	6.50	0.0107664	1.15	2.77	1.74	3.07	2.13	9.23	1.58	4.89
TNFRSF21	1.75	4.42	5.27	0.0216593	7.70	28.19	5.14	5.66	4.62	66.76	15.39	37.22
TNFSF8	1.06	5.13	5.38	0.0203607	18.73	32.05	15.55	26.28	23.95	89.33	26.78	47.81
TREM1	1.14	6.58	5.00	0.0252843	23.60	53.27	104.65	126.07	41.15	198.53	77.50	141.80
TREM2	1.37	4.79	5.60	0.0179386	15.24	26.86	5.69	37.60	41.40	64.76	8.22	21.29
TRIM36	1.10	2.23	4.59	0.0322106	1.50	6.33	5.63	10.39	3.12	4.04	2.08	4.46
TRIM66	1.13	2.21	5.46	0.0194857	1.59	7.69	4.12	4.61	4.70	6.82	1.87	5.67
TSPYL2	1.23	6.17	5.69	0.0170967	24.39	140.15	23.78	47.45	49.41	137.05	77.99	74.47
TTYH2	1.14	5.10	7.00	0.0081329	18.31	58.05	13.19	30.38	21.65	62.10	33.85	37.07
UBD	2.70	5.20	5.75	0.0164654	6.28	155.31	2.20	14.24	4.61	56.32	40.85	15.80
VEGFA	1.73	8.12	7.86	0.0050553	87.57	608.54	75.92	136.50	127.28	456.90	194.72	532.36
VMO1	2.17	3.08	12.07	0.0005122	3.78	19.70	3.60	13.87	1.74	11.86	3.32	9.91
WIPI1	1.15	5.05	4.12	0.0424302	13.62	53.12	7.55	14.27	28.10	85.91	29.99	31.99
YPEL3	1.01	4.69	4.64	0.0312608	24.18	66.76	12.16	15.96	11.59	29.11	16.70	30.49
ZBTB46	1.65	2.34	8.29	0.0039764	2.28	5.99	1.34	5.86	1.61	9.52	5.67	8.15
ZMIZ1	1.00	7.79	5.95	0.0147505	70.77	228.25	208.67	309.09	131.10	333.58	210.74	279.14
ZMYND15	1.26	4.02	6.32	0.0119295	4.81	19.36	18.91	30.61	5.03	22.18	13.44	15.32
ZNF358	1.18	2.52	4.36	0.0368616	3.87	12.92	1.61	3.29	2.10	8.43	7.18	6.68
ZNF395	1.28	4.13	8.10	0.004438	6.68	32.36	8.37	16.54	11.07	22.44	15.06	27.73
ZNF426	1.02	1.73	4.74	0.0294102	3.01	4.66	1.23	3.46	2.02	6.48	2.58	3.10
ZNF766	1.10	2.01	5.41	0.0200007	2.61	7.62	1.27	3.83	2.73	4.64	4.01	5.68
41155	-1.52	4.05	9.24	0.0023616	24.62	13.34	21.71	8.02	22.87	3.40	25.81	12.45
AMER1	-1.82	2.23	8.75	0.0030906	3.83	1.40	11.95	1.64	5.21	1.76	8.16	3.02
AMIGO2	-1.24	2.28	7.52	0.006107	7.26	3.33	8.12	2.52	6.82	2.92	4.89	2.58
ANKRD22	-1.04	7.49	7.10	0.0077046	227.80	140.00	254.99	175.23	237.82	70.51	228.70	100.47
BOLA3	-1.27	1.84	6.61	0.0101563	7.13	1.86	4.03	2.84	5.60	1.93	3.27	1.53
BRIX1	-1.06	3.01	6.40	0.0114355	10.17	5.91	14.31	4.76	9.76	3.52	8.92	6.82
C20orf24	-1.03	1.90	4.55	0.032894	7.32	2.92	6.84	2.67	2.93	1.91	3.09	1.73
CASP5	-1.38	4.33	8.49	0.0035642	34.05	16.67	16.11	6.10	32.48	9.12	32.53	13.42
CCDC86	-1.15	4.04	6.66	0.0098649	32.27	12.88	22.83	13.72	16.44	10.36	17.55	4.73
CCL8	-1.44	7.43	5.42	0.0199475	230.70	94.23	90.14	80.04	217.63	111.63	510.84	43.02
CD274	-1.12	6.94	6.54	0.0105194	113.39	46.26	153.17	154.53	182.25	68.00	204.67	62.04
CMPK2	-1.66	6.34	9.11	0.0025437	116.96	36.98	72.76	26.08	107.70	19.42	180.61	87.90
CX3CR1	-1.45	3.34	4.72	0.0297538	28.52	5.95	9.31	1.75	5.00	2.60	14.69	12.90
CXCL10	-1.71	9.79	6.91	0.008553	1211.64	399.51	1121.31	717.56	518.16	144.65	2601.03	386.25
DOLPP1	-1.38	2.34	7.62	0.0057724	10.08	4.55	6.00	2.77	6.82	1.58	5.84	2.59
EDN1	-1.21	4.45	3.89	0.0486204	32.09	15.69	26.87	47.70	17.71	3.90	25.25	5.22
ETV7	-1.30	4.32	6.51	0.0106973	26.76	4.09	41.12	21.14	16.77	9.05	25.56	15.00
FAHD1	-1.04	3.06	5.42	0.0198604	14.44	4.70	8.49	2.57	12.11	7.93	8.58	7.30
FDX1L	-1.05	1.70	4.99	0.0254416	6.15	2.24	4.56	2.92	3.30	1.11	3.15	2.18
FFAR2	-1.57	5.10	6.52	0.0106658	50.23	8.07	36.96	32.57	21.18	6.57	90.62	27.46
FZD5	-1.10	3.29	6.75	0.0093507	15.82	4.13	13.07	8.59	14.03	5.35	9.72	6.83
GAPT	-1.12	4.33	4.36	0.0367633	36.34	12.05	16.29	8.09	28.03	4.98	20.82	34.32

GIMAP7	-1.35	5.26	10.14	0.0014541	47.95	18.57	68.16	28.15	44.66	15.40	58.65	25.33
GIMAP8	-1.01	5.48	5.86	0.0154615	35.70	37.09	46.67	21.55	87.82	20.83	69.55	36.91
GMPR	-1.18	3.19	5.72	0.0167438	12.20	8.11	6.14	2.21	13.41	5.35	18.01	7.17
GPBAR1	-1.12	4.45	5.49	0.0191745	40.58	16.18	37.53	16.24	18.39	4.73	20.26	21.03
GRIN3A	-1.39	3.67	9.03	0.0026569	13.54	5.95	25.71	9.37	18.14	6.99	15.83	5.50
HERC5	-1.04	4.85	4.84	0.0277677	28.23	17.39	27.68	9.72	27.64	12.99	68.88	38.00
IFIH1	-1.12	6.30	6.84	0.0089033	86.84	57.71	66.35	47.27	108.74	36.63	176.24	49.87
IFIT1	-1.74	4.70	5.46	0.0194801	25.38	8.87	8.06	3.43	37.12	5.71	88.06	30.52
IFIT2	-1.84	7.42	9.72	0.0018271	339.18	75.78	129.51	99.25	240.13	39.01	365.99	85.30
IFIT3	-1.98	7.67	11.38	0.000741	325.02	54.71	249.20	79.92	229.57	60.22	487.02	141.49
IFIT5	-1.17	3.64	4.96	0.0258846	21.49	2.42	14.89	7.05	9.22	8.88	21.42	13.78
IFITM1	-1.12	7.50	4.13	0.0422003	249.82	176.15	79.02	46.62	267.30	56.08	384.36	191.31
IPCEF1	-1.03	3.79	5.64	0.0175994	14.92	10.08	22.88	13.05	24.04	5.38	11.96	7.82
ISG15	-1.29	6.22	7.30	0.0068873	147.14	52.51	47.24	36.36	91.83	29.37	144.90	46.35
ISG20	-1.00	5.46	6.42	0.0112658	54.27	34.52	43.96	27.42	45.71	18.01	91.59	36.58
LY6E	-1.00	8.10	4.96	0.025933	422.24	239.54	183.93	117.38	324.90	110.81	526.13	263.73
MAP3K7CL	-1.40	4.94	7.34	0.0067462	30.03	19.97	64.34	29.21	41.59	18.78	35.48	5.12
MINA	-1.79	2.39	10.78	0.0010247	7.42	2.16	6.06	2.39	9.02	2.58	9.82	2.07
MRPL1	-1.42	1.73	8.36	0.0038391	4.36	1.71	5.82	2.09	5.08	2.09	3.64	1.22
MRPS26	-1.03	2.02	4.72	0.0297303	8.01	2.88	6.54	3.64	3.14	2.04	4.00	1.76
MX1	-1.45	8.78	8.37	0.0038071	702.43	240.18	378.06	168.33	621.25	147.77	833.81	415.83
NME1	-1.11	2.91	6.26	0.0123523	14.41	3.52	8.74	5.60	10.74	4.07	7.08	5.37
NMI	-1.02	6.33	7.63	0.0057355	115.91	43.57	107.50	73.22	91.70	34.62	110.08	66.75
NOC3L	-1.10	2.38	5.77	0.0163416	4.81	1.40	8.39	4.08	7.17	3.99	7.11	4.14
OAS1	-1.11	7.12	6.58	0.010326	222.32	95.06	105.16	67.32	172.29	59.84	262.36	128.48
OAS2	-1.27	7.94	8.49	0.0035724	347.68	120.19	267.18	120.26	336.93	112.37	413.87	239.22
OAS3	-1.42	9.08	7.84	0.005117	803.55	271.52	451.41	216.10	793.23	175.90	1045.75	557.38
P2RY13	-1.73	4.20	8.27	0.0040366	28.39	7.16	47.38	16.56	12.11	3.05	23.01	8.59
P2RY6	-1.07	4.63	6.21	0.0126853	52.77	26.83	23.63	10.33	27.11	11.14	29.79	16.83
PDLIM2	-1.02	3.93	6.57	0.0103855	25.50	13.64	18.20	12.45	15.84	6.80	21.26	7.94
PDSS1	-1.09	2.86	5.30	0.0213308	9.49	5.19	7.05	5.08	16.92	3.45	6.64	3.88
PEX3	-1.03	2.18	4.68	0.0305035	6.25	1.21	4.19	3.84	7.29	4.81	5.55	2.52
PLAC8	-1.95	5.88	9.11	0.0025404	131.59	24.33	54.53	14.02	126.78	13.52	52.88	51.78
POLR3D	-1.09	4.15	6.58	0.0103101	30.16	12.73	34.16	14.05	16.83	9.95	16.32	7.68
PPM1K	-1.34	2.74	4.48	0.034244	6.30	4.93	3.40	3.76	16.01	1.56	13.62	3.57
PSMG1	-1.04	2.91	5.80	0.0160487	12.02	2.42	10.00	6.43	9.46	5.38	8.10	5.68
PVT1	-1.09	1.81	4.56	0.032815	2.81	1.21	2.92	1.55	5.13	2.26	7.92	3.74
RRP9	-1.01	3.17	6.72	0.0095392	14.77	5.30	11.36	6.87	11.63	6.57	10.06	4.89
RSAD2	-2.16	7.07	9.51	0.002038	176.01	39.06	75.74	30.18	236.11	27.25	393.50	95.82
SIGLEC11	-1.13	5.83	7.29	0.0069452	94.49	49.41	75.51	36.54	94.79	40.00	45.17	18.44
SLC6A12	-1.03	5.02	5.33	0.0209586	37.34	17.01	71.92	40.37	33.07	11.28	29.36	19.36
SMYD5	-1.02	3.27	5.02	0.0250475	15.98	2.92	16.59	9.07	9.65	7.37	8.83	6.27
SOCS1	-1.14	2.42	6.02	0.0141718	9.38	4.40	5.82	3.61	9.39	2.83	4.71	2.27
SPRYD7	-1.03	1.42	5.21	0.022465	4.78	1.97	2.54	1.49	3.62	2.33	3.18	1.18
TACO1	-1.22	3.01	5.63	0.0176535	10.19	3.07	14.14	10.16	9.97	6.72	7.78	1.79
TIFA	-1.58	4.84	8.35	0.0038593	38.95	10.53	73.98	28.91	32.48	11.11	24.12	8.29
TMA16	-1.40	2.40	7.81	0.0051887	8.76	2.39	9.32	1.94	5.31	3.28	6.79	3.95
TNFSF10	-1.28	7.61	7.51	0.0061487	361.52	184.79	229.78	116.10	175.24	51.37	323.94	123.88
USP18	-2.14	5.33	7.87	0.005033	63.09	25.39	13.44	3.17	64.95	7.32	115.50	28.02
UTP20	-1.31	3.27	7.51	0.0061251	9.99	5.00	17.53	10.04	15.11	3.45	11.25	4.52
WARS2	-1.12	1.79	5.80	0.0159982	4.31	1.82	4.40	3.59	4.88	1.14	4.59	2.51
XPA	-1.01	3.41	5.87	0.0154363	18.41	5.95	9.48	5.51	13.15	5.76	15.21	11.08

Table S2. Functional enrichment pathways in treatment-specific signature genes.

Category	Term	Count	%	PValue	Genes	Fold Enrichm	Bonferroni	Benjamini	FDR
GOTERM_BP_ALL	GO:0002376~immune system process	33	15.27778	1.39E-06	IFIH1, MMP9, EDN1, OAS3, RSAD2, CCL8, ACP5, OAS1, IL32, OAS2, CXCI	2.55060942	0.00230315	0.00230315	0.00234
GOTERM_BP_ALL	GO:0042221~response to chemical stimulus	37	17.12963	5.9064E-06	A2M, SLC15A2, EDN1, PDE3B, ACP5, CCL8, FOXO1, HSPA1A, GRIN3A, H!	2.22798957	0.00973919	0.00488151	0.00993
GOTERM_BP_ALL	GO:0006955~immune response	25	11.57407	8.9681E-06	IFIH1, OAS3, CCL8, ACP5, RSAD2, IL32, OAS1, OAS2, CXCL10, HLA-DOA,	2.79480478	0.01475042	0.00494118	0.01508
GOTERM_BP_ALL	GO:0050896~response to stimulus	71	32.87037	2.8626E-05	PPARD, A2M, NUAK2, SLC15A2, EDN1, PDE3B, FOXO1, GRIN3A, CXCL10	1.56387763	0.0463271	0.0117886	0.04813
GOTERM_BP_ALL	GO:0006950~response to stress	42	19.44444	3.8647E-05	IFIH1, PPARD, A2M, NMI, NUAK2, EDN1, CCL8, RSAD2, IL32, HSPA1A, H	1.922693	0.06203129	0.01272607	0.06497
GOTERM_BP_ALL	GO:0001568~blood vessel development	13	6.018519	8.0439E-05	SHB, EPAS1, APOE, TNFRSF12A, ZMIZ1, VEGFA, EDN1, MMP19, FOXO1,	4.09296309	0.12479076	0.02197044	0.13517
GOTERM_BP_ALL	GO:0048514~blood vessel morphogenesis	12	5.555556	9.2519E-05	SHB, EPAS1, TNFRSF12A, APOE, ZMIZ1, VEGFA, EDN1, MMP19, ANPEP,	4.38691632	0.14213693	0.02166344	0.15546
GOTERM_BP_ALL	GO:0001944~vasculature development	13	6.018519	0.00010156	SHB, EPAS1, APOE, TNFRSF12A, ZMIZ1, VEGFA, EDN1, MMP19, FOXO1,	3.99512333	0.15489041	0.0208164	0.17064
GOTERM_BP_ALL	GO:0006952~defense response	20	9.259259	0.00037558	A2M, IFIH1, NMI, C5AR1, SOCS6, RSAD2, CCL8, IL32, COLEC12, NLRP1, (	2.50850771	0.46337107	0.06682351	0.62968
GOTERM_BP_ALL	GO:0001525~angiogenesis	9	4.166667	6.65E-04	SHB, EPAS1, TNFRSF12A, VEGFA, EDN1, MMP19, ANPEP, MMP14, THBS	4.69073992	0.66794531	0.10438582	1.11255
GOTERM_BP_ALL	GO:0030335~positive regulation of cell migration	7	3.240741	0.00101412	CXCL16, MMP9, VEGFA, EDN1, HBEGF, THBS1, CXCL10	6.06692454	0.81385985	0.14173417	1.69168
GOTERM_BP_ALL	GO:0032101~regulation of response to external stimulus	9	4.166667	0.00106245	A2M, SERPINF1, APOE, VEGFA, EDN1, SERPINE1, GRIN3A, THBS1, SPP1	4.36622332	0.82819959	0.13652053	1.77163
GOTERM_BP_ALL	GO:0040017~positive regulation of locomotion	7	3.240741	0.00167024	CXCL16, MMP9, VEGFA, EDN1, HBEGF, THBS1, CXCL10	5.509758	0.93733158	0.19189966	2.77178
GOTERM_BP_ALL	GO:0051272~positive regulation of cell motion	7	3.240741	0.00167024	CXCL16, MMP9, VEGFA, EDN1, HBEGF, THBS1, CXCL10	5.509758	0.93733158	0.19189966	2.77178
GOTERM_BP_ALL	GO:0042219~cellular amino acid derivative catabolic process	4	1.851852	0.00175462	COLQ, SMOX, PCYOX1, AFMID	16.2392867	0.94552212	0.18767463	2.90988
GOTERM_BP_ALL	GO:0048519~negative regulation of biological process	38	17.59259	0.00282406	PPARD, A2M, IFITM1, NUAK2, HSD3B7, LGMN, EDN1, APOC1, PDE3B, F	1.61765522	0.99077728	0.26831539	4.64418
GOTERM_BP_ALL	GO:0040012~regulation of locomotion	9	4.166667	0.00346305	BBS1, APOE, CXCL16, MMP9, VEGFA, EDN1, HBEGF, THBS1, CXCL10	3.61577869	0.99681158	0.30181031	5.66648
GOTERM_BP_ALL	GO:0051270~regulation of cell motion	9	4.166667	0.00357116	BBS1, APOE, CXCL16, MMP9, VEGFA, EDN1, HBEGF, THBS1, CXCL10	3.59704408	0.99733622	0.29439983	5.83842
GOTERM_BP_ALL	GO:0009611~response to wounding	16	7.407407	0.00362032	A2M, PPARD, NMI, CCL8, CCL18, CXCL10, CD9, THBD, TFRC, SERPINE1, (	2.32865244	0.9975453	0.28385667	5.9165
GOTERM_BP_ALL	GO:0010594~regulation of endothelial cell migration	4	1.851852	0.00393512	APOE, VEGFA, EDN1, THBS1	12.3418579	0.99854588	0.29097239	6.41509
GOTERM_BP_ALL	GO:0010033~response to organic substance	19	8.796296	0.00555163	A2M, BAIAP2, SOCS1, FOXO1, ACP5, PDE3B, HSPA1A, COLEC12, GRIN3/	2.03272625	0.99990142	0.36949507	8.93648
GOTERM_BP_ALL	GO:0015758~glucose transport	4	1.851852	0.00602047	PPARD, SLC2A3, SLC2A1, EDN1	10.6395327	0.99995487	0.37903423	9.65571
GOTERM_BP_ALL	GO:0030334~regulation of cell migration	8	3.703704	0.00632906	APOE, CXCL16, MMP9, VEGFA, EDN1, HBEGF, THBS1, CXCL10	3.65143726	0.99997302	0.38010668	10.1262
GOTERM_BP_ALL	GO:0008645~hexose transport	4	1.851852	0.00662677	PPARD, SLC2A3, SLC2A1, EDN1	10.2848816	0.99998358	0.3806004	10.5779
GOTERM_BP_ALL	GO:0009605~response to external stimulus	22	10.18519	0.00715848	A2M, PPARD, NMI, C5AR1, NUAK2, CCL8, GRIN3A, MMP14, CCL18, CXC	1.85667994	0.99999324	0.39104517	11.3794
GOTERM_BP_ALL	GO:0015749~monosaccharide transport	4	1.851852	0.00726813	PPARD, SLC2A3, SLC2A1, EDN1	9.95311123	0.99999437	0.38337295	11.5438
GOTERM_BP_ALL	GO:0048523~negative regulation of cellular process	34	15.74074	0.00734041	A2M, PPARD, IFITM1, NUAK2, HSD3B7, EDN1, APOC1, PDE3B, FOXO1, I	1.57990651	0.99999501	0.37470733	11.652
GOTERM_BP_ALL	GO:0048585~negative regulation of response to stimulus	6	2.777778	0.00947671	A2M, SERPINF1, APOE, SOCS1, GRIN3A, SPP1	4.62819672	0.99999986	0.44253874	14.7954

Category	Term	Count	%	PValue	Genes	Fold Enrichm	Bonferroni	Benjamini	FDR
PANTHER_BP_ALL	BP00156:Interferon-mediated immunity	9	4.166667	7.8868E-07	IFIT3, IFIT2, IFIT1, NMI, IFIT5, OAS3, OAS1, OAS2, CXCL10	11.8297146	1.08E-04	1.08E-04	9.27E-04
PANTHER_BP_ALL	BP00148:Immunity and defense	36	16.66667	1.1555E-06	PHLPP1, A2M, PPARD, NMI, IFITM1, OAS3, CCL8, ITGB5, OAS1, HSPA1A	2.40472888	1.58E-04	7.92E-05	0.00136
PANTHER_BP_ALL	BP00071:Proteolysis	19	8.796296	0.01810289	A2M, FBXL20, MMP9, LGMN, MMP19, HERC5, ANPEP, MMP14, CASP5,	1.79003261	0.91814613	0.56581007	19.3244
PANTHER_BP_ALL	BP00122:Ligand-mediated signaling	11	5.092593	0.01984852	A2M, PPARD, TNFSF10, LILRB5, VEGFA, EDN1, SOCS6, CCL8, HBEGF, CCI	2.32235618	0.93585421	0.49674044	20.9942
PANTHER_BP_ALL	BP00176:Blood clotting	5	2.314815	0.02167494	CD9, THBD, ITGB5, THBS1, THBS3	4.68353964	0.95031842	0.45142112	22.7074
PANTHER_BP_ALL	BP00116:JNK cascade	4	1.851852	0.03670277	SH3BP5, TNFRSF21, DUSP4, CXCL10	5.43290598	0.99404115	0.57421164	35.5659
PANTHER_BP_ALL	BP00114:MAPKKK cascade	6	2.777778	0.06551715	SPRY2, DUSP4, ETV7, RPS6KA2, PPM1K, VEGFA	2.76249457	0.99990704	0.7345156	54.9089
PANTHER_BP_ALL	BP00107:Cytokine and chemokine mediated signaling pathwa	7	3.240741	0.06573994	TNFSF10, SOCS1, CX3CR1, SOCS6, CCL8, CCL18, CXCL10	2.44830527	0.99991003	0.68792371	55.0351
PANTHER_BP_ALL	BP00145:Small molecule transport	5	2.314815	0.07790946	SLC15A2, SLC6A8, SLC6A12, SLC25A29, ABCC3	3.0868784	0.99998507	0.70907876	61.4572
